# ERF Transcription Factor OsBIERF3 Positively Contributes to Immunity against Fungal and Bacterial Diseases but Negatively Regulates Cold Tolerance in Rice

**DOI:** 10.3390/ijms23020606

**Published:** 2022-01-06

**Authors:** Yongbo Hong, Hui Wang, Yizhou Gao, Yan Bi, Xiaohui Xiong, Yuqing Yan, Jiajing Wang, Dayong Li, Fengming Song

**Affiliations:** 1 State Key Laboratory of Rice Biology, Institute of Biotechnology, College of Agriculture and Biotechnology, Zhejiang University, Hangzhou 310058, China; yongbohong@126.com (Y.H.); whwanghui@zju.edu.cn (H.W.); 11616060@zju.edu.cn (Y.G.); 11716065@zju.edu.cn (Y.B.); 289963@zju.edu.cn (X.X.); 11816061@zju.edu.cn (Y.Y.); 11816055@zju.edu.cn (J.W.); dyli@zju.edu.cn (D.L.); 2 State Key Laboratory of Rice Biology, China National Rice Research Institute, Hangzhou 310006, China

**Keywords:** rice (*Oryza sativa* L.), ERF transcription factor, *OsBIERF3*, disease resistance, cold tolerance

## Abstract

We previously showed that overexpression of the rice ERF transcription factor gene *OsBIERF3* in tobacco increased resistance against different pathogens. Here, we report the function of *OsBIERF3* in rice immunity and abiotic stress tolerance. Expression of *OsBIERF3* was induced by *Xanthomonas oryzae* pv. *oryzae*, hormones (e.g., salicylic acid, methyl jasmonate, 1-aminocyclopropane-1-carboxylic acid, and abscisic acid), and abiotic stress (e.g., drought, salt and cold stress). OsBIERF3 has transcriptional activation activity that depends on its C-terminal region. The *OsBIERF3*-overexpressing (OsBIERF3-OE) plants exhibited increased resistance while *OsBIERF3*-suppressed (OsBIERF3-Ri) plants displayed decreased resistance to *Magnaporthe oryzae* and *X. oryzae* pv. *oryzae*. A set of genes including those for PRs and MAPK kinases were up-regulated in OsBIERF3-OE plants. Cell wall biosynthetic enzyme genes were up-regulated in OsBIERF3-OE plants but down-regulated in OsBIERF3-Ri plants; accordingly, cell walls became thicker in OsBIERF3-OE plants but thinner in OsBIERF3-Ri plants than WT plants. The OsBIERF3-OE plants attenuated while OsBIERF3-Ri plants enhanced cold tolerance, accompanied by altered expression of cold-responsive genes and proline accumulation. Exogenous abscisic acid and 1-aminocyclopropane-1-carboxylic acid, a precursor of ethylene biosynthesis, restored the attenuated cold tolerance in OsBIERF3-OE plants while exogenous AgNO_3_, an inhibitor of ethylene action, significantly suppressed the enhanced cold tolerance in OsBIERF3-Ri plants. These data demonstrate that OsBIERF3 positively contributes to immunity against *M. oryzae* and *X. oryzae* pv. *oryzae* but negatively regulates cold stress tolerance in rice.

## 1. Introduction

As sessile organisms, plants are unable to escape from unfavorable environments and thus often suffer from numerous abiotic (drought, salt, extreme temperature, etc.) and biotic (pathogens, herbivore insects, etc.) stresses. To cope with external stresses and survive, plants have developed multi-layered and fine-toned mechanisms at molecular, biochemical, physiological, metabolic, and developmental levels [1,2,3,4,5,6]. Upon perception of external stress signals, complicated hormone-mediated signaling networks are often activated in effective and timely manners [7,8,9,10,11], which ultimately lead to transcriptional reprogramming that coordinately regulates the expression of a large set of genes to initiate stress responses [12,13,14,15]. This large-scale transcriptional reprogramming of gene expression in a specific stress response requires the concerted action of chromatin dynamics and different types of transcription factors (TFs) in both temporal and spatial manners [15,16,17,18,19,20].

Genetic studies have demonstrated that dozens of TFs from the families of WRKY, AP2/ERF (Apetala2/Ethylene Responsive Factor), NAC (NAM, ATAF and CUC), bZIP (basic leucine zipper domain), bHLH (basic helix-loop-helix), and Myb play crucial roles in modulating biotic and abiotic stress responses in higher plants [16,21,22,23,24,25,26,27,28,29]. The AP2/ERF superfamily is a large plant-specific TF family and the rice AP2/ERF superfamily consists of 163 members, among which 134 belong to the ERF subfamily [30,31]. ERF proteins typically possess at least one AP2/ERF domain consisting of 58 or 59 conserved amino acid residues and can bind specifically to DNA sequences containing GCC and/or DRE/CRT boxes [32,33,34]. ERFs act as either transcription activators or repressors that can activate or repress the transcription of stress-responsive genes, whose promoters harbor at least one core GCC box [32,35,36]. For example, Arabidopsis AtERF1, AtERF2 and AtERF5 are activators, while AtERF3, AtERF4 and AtERF7 are repressors [37]. ERFs have been shown to participate in diverse biological processes including plant growth and development, immunity, and abiotic stress tolerance [22,27,28,38,39,40,41,42] and therefore provide promising potential in the improvement of biotic and abiotic stress tolerance in crop plants [43,44,45].

The importance of ERFs in plant immunity has been extensively explored through overexpression and knockout/knockdown approaches in Arabidopsis, rice, and other plants [22,43]. In rice, infection of *Magnaporthe oryzae*, the causal agent of blast disease, or elicitor treatment induced the expression of *OsEREBP1*, *OsBIERF1*, *OsBIERF3*, *OsBIERF4*, *OsERF83*, *OsERF922*, and *OsEBP2* [46,47,48,49,50]. Overexpression of the *M. oryzae*-induced *OsBIERF3* in tobacco or the cell-wall-degrading enzyme-induced *OsAP2/ERF152* in Arabidopsis conferred increased resistance against fungal, bacterial, or viral pathogens [51,52]. Transgenic rice plants overexpressing *OsERF83* exhibited a significant enhancement of resistance against *M. oryzae*, accompanied by the up-regulated expression of defense genes [50]. Knockdown of *OsERF922* activated the expression of defense genes and enhanced resistance against *M. oryzae,* while the *OsERF922*-overexpressing plants showed reduced expression of defense genes and enhanced susceptibility to *M. oryzae*, indicating that OsERF922 is a negative regulator of rice immunity against *M. oryzae* [48,53]. Differential dynamics of the regulatory network topology showed that ERFs (e.g., ERF104, ERF83, ERF91, ERF118, and ERF47) play a crucial role during signal crosstalk in rice plants responding to *Xanthomonas oryzae* pv. *oryzae*, the causal agent of bacterial leaf blight disease, under high or low temperature [54]. OsEREBP1, phosphorylated by OsMPK12, exhibited enhanced binding to the GCC box element of defense gene promoters [55], and overexpression of *OsEREBP1* enhanced resistance against *X. oryzae* pv. *oryzae* [56,57]. Functional analysis of African *X. oryzae* pv. *oryzae* TALomes revealed that OsERF123 is a new bacterial blight susceptibility gene in rice [58]. Furthermore, feeding by rice striped stem borer (*Chilo suppressalis*) significantly and rapidly up-regulated the expression of *OsERF3,* and functional studies revealed that *OsERF3* regulates rice resistance to this insect pest through affecting early components of herbivore-induced defense responses [59].

ERFs have also been reported to play critical roles in plant response to different abiotic stresses, such as drought, submergence, high salinity, and extreme temperature [27,28,38,39,40]. In rice, Sub1A and SNORKEL1/2 modulate flooding tolerance via two different physiological mechanisms [60,61,62,63]. Overexpression of *OsDREB1s*, *OsDREB2A*, *OsEREBP1*, *OsERF48*, *OsERF71*, *OsLG3*, *OsERF83*, *OsERF101*, *OsERF115*, and *OsERF4a* (*OsERF3*) improved drought tolerance in rice, through activating the jasmonate and abscisic acid (ABA) signaling pathways or modulating root architecture [57,64,65,66,67,68,69,70,71,72,73,74,75,76,77,78]. By contrast, *OsEBP89*, *OsERF109*, *OsDERF1*, *OsERF3,* and *OsAP2-39* negatively regulated rice drought tolerance [70,79,80,81,82]. Overexpression of *OsDREB1F*, *OsDREB2A*, *OsDRAP1*, *OsERF19,* or *OsSTAP1* improved salt tolerance in transgenic rice, and the amino acid and carbohydrate metabolism pathways play crucial roles in *OsDRAP1*-mediated salt tolerance [66,68,83,84,85,86]. OsERF922 and OsERF106 are negative regulators of rice salt tolerance [48,87], probably through modulation of ABA level [48]. OsEREBP1 and OsEREBP2 negatively regulate the expression of *OsRMC*, encoding a receptor-like kinase that is a negative regulator of salt stress responses in rice [88]. Some rice *OsERF* genes, such as *OsDREB1s*, *OsEREBP1*, *OsSTAP1* and *OsAP25*, were reported to be induced under low temperature or cold stress conditions [46,54,64,86,88,89,90,91], and overexpression of *OsDREB1A*, *OsDREB1F* or *OsDREB1G* in transgenic rice increased tolerance to cold or low-temperature stress [66,90,92]. Recently, it was found that the overexpression of *OsERF115* conferred enhanced heat tolerance in transgenic rice plants [78].

In our previous studies, we found that *OsBIERF3* was induced by *M. oryzae* and ectopic overexpression of *OsBIERF3* in tobacco increased resistance to bacterial and viral diseases [46,51]. However, the function of *OsBIERF3* in rice immunity is yet unknown. In the present study, we generated OsBIERF3-OE and OsBIERF3-Ri transgenic rice lines with overexpression or RNAi-mediated suppression of the endogenous *OsBIERF3* gene and evaluated their resistance against fungal and bacterial pathogens, as well as their abiotic stress tolerance. Our phenotyping, molecular and biochemical analyses demonstrate that OsBIERF3 positively contributes to immunity against *M. oryzae* and *X. oryzae* pv. *oryzae* through affecting the MAPK cascade and cell wall biosynthesis pathways, but negatively regulates cold tolerance in rice.

## 2. Results

### 2.1. Responsiveness of OsBIERF3 to a Bacterial Pathogen, Abiotic Stress, and Hormones

**Figure 1:**
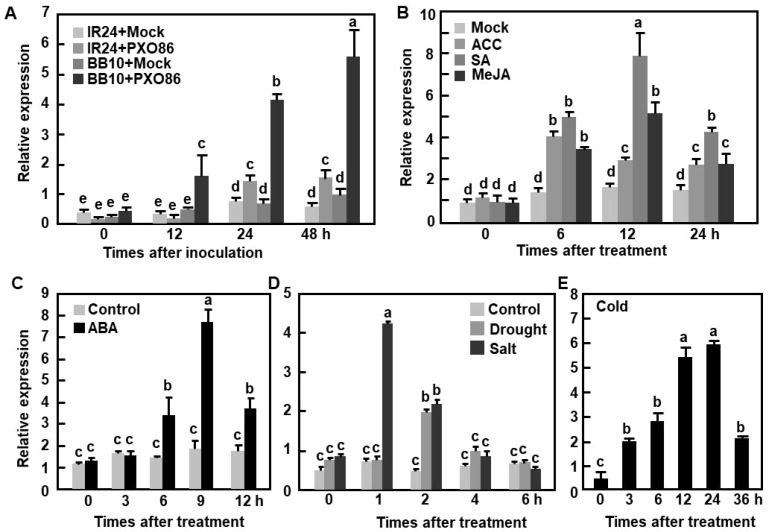
Responsiveness of *OsBIERF3* to *Xanthomonas oryzae* pv. *oryzae*, abiotic stress, and hormones. (**A**) Expression of *OsBIERF3* in incompatible and compatible rice-*X. oryzae* pv. *oryzae* interactions. Rice plants of IR24 and BB10 were inoculated with *X. oryzae* pv. *oryzae* strain PXO86 and mock-inoculated plants were used as controls. (**B**,**C**) Expression of *OsBIERF3* in rice plants treated with different defense signaling hormones. Two-week-old rice plants of cv. Yuanfengzo were foliar sprayed with 100 μM MeJA, 100 μM ACC, 150 μM SA, 100 μM ABA or sterilized distilled water as controls. (**D**) Expression of *OsBIERF3* in drought- and salt-treated rice plants. Drought stress was applied by placing detached leaves on lab benches without water supply. Salt stress was applied by irrigation with 150 mM NaCl. (**E**) Expression of *OsBIERF3* in cold-stressed rice plants. Cold stress was applied by transferring rice plants of cv. Yuanfengzao to a 4 °C growth chamber. Leaf samples were collected at indicated time points for qRT-PCR analyses of gene expression. Fold relative expression levels as compared to those of the *Actin* gene are presented as the means ± SD from three independent experiments and different letters indicate statistically significant difference at *p* < 0.05 level.

We previously observed that *OsBIERF3* was induced by benzothiadiazole, an analog of salicylic acid (SA), and by *M. oryzae* [46]. We further examined the responsiveness of *OsBIERF3* in response to *X. oryzae* pv. *oryzae* by analyzing the transcript levels in the incompatible and compatible interactions between a pair of rice near-isogenic lines IR24 and BB10 and *X. oryzae* pv. *oryzae*. Rice variety IR24 lacks the *Xa10* gene and gives a compatible response to *X. oryzae* pv. *oryzae* strain PXO86, carrying the corresponding *avrXa10* gene, while its near-isogenic line BB10 harbors the *Xa10* gene and confers an incompatible response to the same strain [93,94]. In an incompatible interaction between rice BB10 and *X. oryzae* pv. *oryzae* PXO86, the transcript level of *OsBIERF3* in inoculated plants started to increase at 12 h post inoculation (hpi) and gradually increased over a period of 48 h, leading to increases of 3.8-, 5.4-, and 5.8-fold, over those in mock-inoculated plants (Figure 1A). By contrast, in a compatible interaction between rice IR24 and *X. oryzae* pv. *oryzae* PXO86, the transcript level of *OsBIERF3* increased at 24 hpi and displayed 1.3- and 2.1-fold increases at 24 and 48 hpi over those in mock-inoculated plants (Figure 1A). These results indicate that *OsBIERF3* responds to *X. oryzae* pv. *oryzae* infection and the *X. oryzae* pv. *oryzae*-induced expression of *OsBIERF3* is much greater during earlier stages of the incompatible interaction than that in the compatible interaction.

The responsiveness of *OsBIERF3* in rice plants of cv. Yuanfengzao to stress hormones, such as ET, SA, jasmonic acid (JA), and ABA, was also examined. Generally, the transcript level of *OsBIERF3* started to increase at 6 h post treatment (hpt) and was maintained at relatively higher levels in rice plants after treatment with 1-aminocyclopropane-1-carboxylic acid (ACC, a precursor of ET biosynthesis), SA, methyl jasmonate (MeJA) or ABA (Figure 1B,C). Particularly, the highest level of *OsBIERF3* transcript in ACC-treated plants was seen at 6 hpt, giving 2.45-fold higher over that in control plants, while the highest levels in SA- and MeJA-treated plants were observed at 12 hpt, showing 4.35- and 2.52-fold higher than that in control plants (Figure 1B). In ABA-treated plants, the *OsBIERF3* transcript peaked, being 3.42-fold higher than that in control plants at 9 hpt (Figure 1C). These data indicate that *OsBIERF3* responds to multiple stress hormones.

The responsiveness of *OsBIERF3* in rice plants of cv. Yuanfengzao to abiotic stress such as drought, salt and cold treatment was further examined. The transcript level of *OsBIERF3* in detached leaves was significantly and rapidly up-regulated within 2 h by fast dehydration, giving a 3.12-fold increase over that in control plants at 2 hpt (Figure 1D). An increase of 4.55-fold in *OsBIERF3* transcript was observed at 1 hpt in NaCl-treated plants but the transcript level decreased to basal level at 4 hpt (Figure 1D). Similarly, *OsBIERF3* transcript level in cold (4 °C)-stressed plants gradually increased and peaked at 24 hpt, showing an 11.42-fold increase over that in control plants (Figure 1E). These results suggest that *OsBIERF3* is an abiotic stress-responsive rice ERF gene.

### 2.2. OsBIERF3 Is a Transcriptional Activator

**Figure 2:**
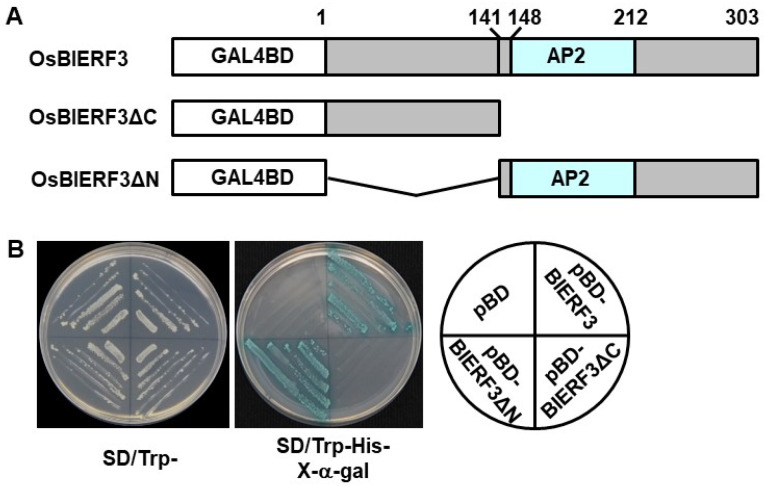
OsBIERF3 is a transcriptional activator. (**A**) Diagrams showing structural feature and deletion mutants of OsBIERF3. (**B**) OsBIERF3 has transactivation activity. Yeast cells carrying pBD-OsBIERF3, pBD-OsBIERF3△C, pBD-OsBIERF3△N or pBD empty vector (as a negative control) were streaked on SD/Trp^–^ plates (left) or SD/Trp^–^His^–^ plates supplemented with x-α-gal for 3 days at 30 °C. Experiments in (**B**) were repeated three times with similar results.

We previously showed that OsBIERF3 is a nucleus-localized protein and can bind to a synthetic sequence containing the core GCC box element in vitro [46]. To verify whether OsBIERF3 had transcriptional activation activity, the entire OsBIERF3, and its two deletion mutants, OsBIERF3ΔC (an N-terminal fragment lacking the AP2 domain-containing C-terminal 142–303 aa) and OsBIERF3ΔN (an AP2 domain-containing C-terminal fragment lacking the N-terminal 1–141 aa), were examined in yeast for their transcriptional activity (Figure 2A). Yeast transformants harboring OsBIERF3 and its deletion mutant constructs grew well on SD/Trp- medium (Figure 2B). On SD/Trp-His-medium, only transformants carrying pBD-OsBIERF3 or pBD-OsBIERF3ΔN grew and showed β-galactosidase activity, whereas yeast transformants carrying pBD-OsBIERF3ΔC and empty vector did not (Figure 2B). These data indicate that OsBIERF3 is a transcriptional activator and its C-terminal region is required for transcription activator activity.

### 2.3. Generation of OsBIERF3-OE and OsBIERF3-Ri Transgenic Lines

To better understand the biological function of OsBIERF3 in rice, we generated OsBIERF3-OE and OsBIERF3-Ri transgenic lines. After screening 27 and 20 independent OsBIERF3-OE and OsBIERF3-Ri lines by hygromycin resistance phenotype on 1/2 MS medium, three independent OsBIERF3-OE (OE-2, OE-4, and OE-6) and three independent OsBIERF3-Ri (Ri-1, Ri-3, and Ri-29) lines were identified as single-copy lines, as confirmed by Southern blotting with a fragment of *HgrII* gene as a probe (Figure S1A). The transcript levels of *OsBIERF3* in plants of T3 generations of stable OsBIERF3-OE lines OE-2, OE-4, and OE-6 were 60.7-, 93.9-, and 28.7-fold higher than that in WT plants, respectively (Figure S1B). By contrast, the transcript levels of *OsBIERF3* in plants of T3 generations of stable OsBIERF3-Ri lines Ri-1, Ri-3, and Ri-29 were approximately 19%, 2%, and 5% of that in WT plants, respectively (Figure S1C). During our studies, we observed growth retardation in OsBIERF3-OE plants at the seedling stage (Figure S2A,C) and this growth retardation phenotype recovered at the adult stage (Figure S2B,E). By contrast, OsBIERF3-Ri plants showed normal growth, as compared with WT plants, at the seedling and adult stages (Figure S2A,B,D,F). Manipulation of *OsBIERF3* in OsBIERF3-OE and OsBIER3-Ri plants had no deleterious impacts on major agronomic traits such as grain yield, the weight of a single panicle and grain numbers per panicle, and even improved some agronomic traits (Figure S3A–F).

### 2.4. OsBIERF3 Positively Regulates Resistance to M. oryzae

**Figure 3:**
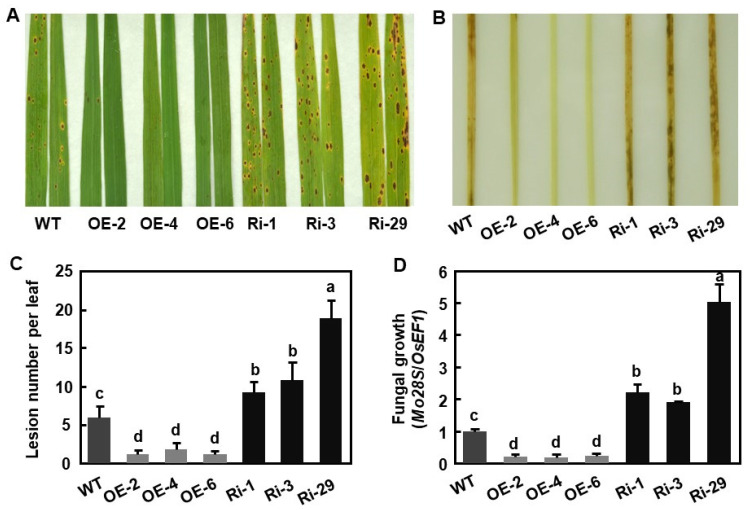
*OsBIERF3* positively regulates resistance against *Magnaporthe oryzae*. (**A**) Representative disease symptom on *M. oryzae*-inoculated leaves. (**B**) Trypan blue staining of dead cells in *M. oryzae*-inoculated leaves at 6 dpi. (**C**) Lesion numbers on inoculated leaves at 7 dpi. (**D**) Quantification of fungal growth in inoculated leaves at 7 dpi. Three-week-old plants were inoculated by foliar spraying with spore suspensions (1 × 10^5^ spores/mL) of *M. oryzae* strain 97-220. At least 30 plants in each of the experiments were evaluated for disease scores using an international nine-scale standard. Amounts of *M. oryzae* 28S rDNA and rice *OsEF1* genomic DNA were estimated by qRT-PCR and relative fungal growth was shown as ratios of *Mo28S*/*OsEF1*. Experiments in (**A**,**B**) were repeated three times with similar results. Data presented in (**C**,**D**) are the means ± SD from three independent experiments and different letters above the columns indicate statistically significant difference at *p* < 0.05 level.

We first evaluated the resistance of OsBIERF3-OE and OsBIERF3-Ri plants against *M. oryzae* by foliar inoculating 3-week-old seedlings with a race ZE3 strain 97-220 of the fungus [95]. Typical *M. oryzae*-caused blast lesions were seen on the inoculated leaves of OsBIERF3-OE, OsBIERF3-Ri, and WT plants; however, the overall blast disease severity on OsBIERF3-OE plants was less severe while the disease severity on OsBIERF3-Ri plants was much more severe, as compared with those in WT plants (Figure 3A). Accordingly, cell death, as revealed by trypan blue staining, in inoculated leaves of OsBIERF3-Ri plants was much heavier, while cell death in inoculated leaves of OsBIERF3-OE plants was less severe, as compared with that in inoculated leaves of WT plants (Figure 3B). At 6 days post inoculation (dpi), the average numbers of the blast lesions on the inoculated leaves of OsBIERF3-Ri plants were increased by 89%, 93%, and 204%, while the numbers of the disease lesions on the inoculated leaves of OsBIERF3-OE plants were decreased by 78%, 69%, and 77%, respectively, as compared with that in WT plants (Figure 3C). Further measurement of in planta fungal growth, as revealed by analyzing the genomic DNA level of the 28S rDNA gene of *M. oryzae*, indicated that OsBIERF3-Ri plants supported more growth of *M. oryzae* in inoculated leaves, leading to increases of 105%, 98%, and 387%, whereas OsBIERF3-OE plants supported less fungal growth, resulting in reductions of 92%, 93%, and 93%, respectively, as compared with that in WT plants (Figure 3D). Together, these results indicate that OsBIERF3-OE plants exhibited an increased resistance while OsBIERF3-Ri displayed an attenuated resistance to *M. oryzae*, and thus *OsBIERF3* is a positive regulator of resistance against *M. oryzae*.

### 2.5. OsBIERF3 Positively Regulates Resistance to X. oryzae pv. oryzae

**Figure 4:**
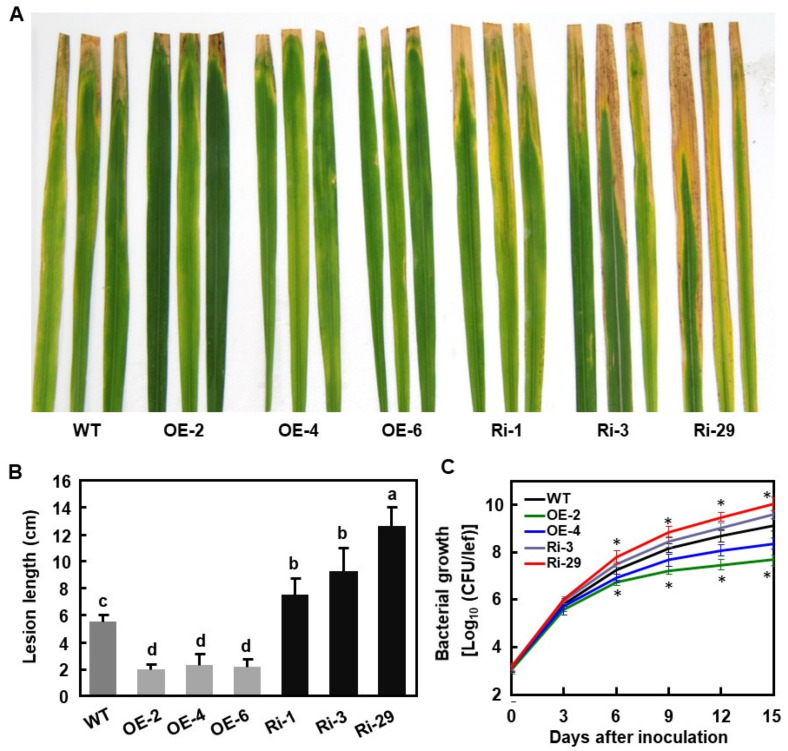
*OsBIERF3* positively regulates resistance against *Xanthomonas oryzae* pv. *oryzae*. Rice plants were inoculated with *X. oryzae* pv. *oryzae* strain PXO86 using the leaf clipping method at the booting stage. (**A**) Disease symptom on the inoculated leaves at 15 dpi. (**B**) Lesion length on the inoculated leaves at 15 dpi. At least 30 plants in each of the experiments were used for the measurement of the lesion lengths. (**C**) Bacterial growth in the inoculated leaves. Leaf samples were collected at indicated time points and bacterial growth was determined from three leaves at each time point. Experiments in (**A**) were repeated three times with similar results. Data presented in (**B**,**C**) are the means ± SD from three independent experiments and different letters in (**B**) and asterisks in (**C**) indicate statistically significant difference at *p* < 0.05 level.

We next evaluated the resistance of OsBIERF3-OE and OsBIERF3-Ri lines against *X. oryzae* pv. *oryzae* by leaf-clipping inoculation of adult plants at booting stage with *X. oryzae* pv. *oryzae* strain PXO86 [94]. The overall *X. oryzae* pv. *oryzae*-caused blight disease on OsBIERF3-OE plants was less severe, while the disease severity on OsBIERF3-Ri plants was more severe, as compared with those in WT plants (Figure 4A). At 15 dpi, the average length of the blight lesions on the inoculated leaves of OsBIERF3-Ri plants were 7.7, 9.2, and 12.7 cm, leading to increases of 40%, 67%, and 131%, while the length of the blight lesions on the inoculated leaves of OsBIERF3-OE plants was 2.0, 2.3, and 2.1 cm, resulting in reductions of 64%, 58%, and 62%, respectively, as compared with that (5.5 cm) in WT plants (Figure 4B). Similarly, the bacterial titers in the inoculated leaves of OsBIERF3-Ri-3 and -29 plants were 3.2- and 7.7-fold higher while the bacterial titers in inoculated leaves of OsBIERF3-OE-2 and -4 plants were 3.6- to 29.2-fold lower than that in WT plants (Figure 4C). These results indicate that OsBIERF3-OE plants exhibited an increased resistance while OsBIERF3-Ri displayed an attenuated resistance to *X. oryzae* pv. *oryzae*, and thus *OsBIERF3* is a positive regulator of resistance against *X. oryzae* pv. *oryzae*.

### 2.6. Identification of Differentially Expressed Genes in OsBIERF3-OE Plants

**Figure 5:**
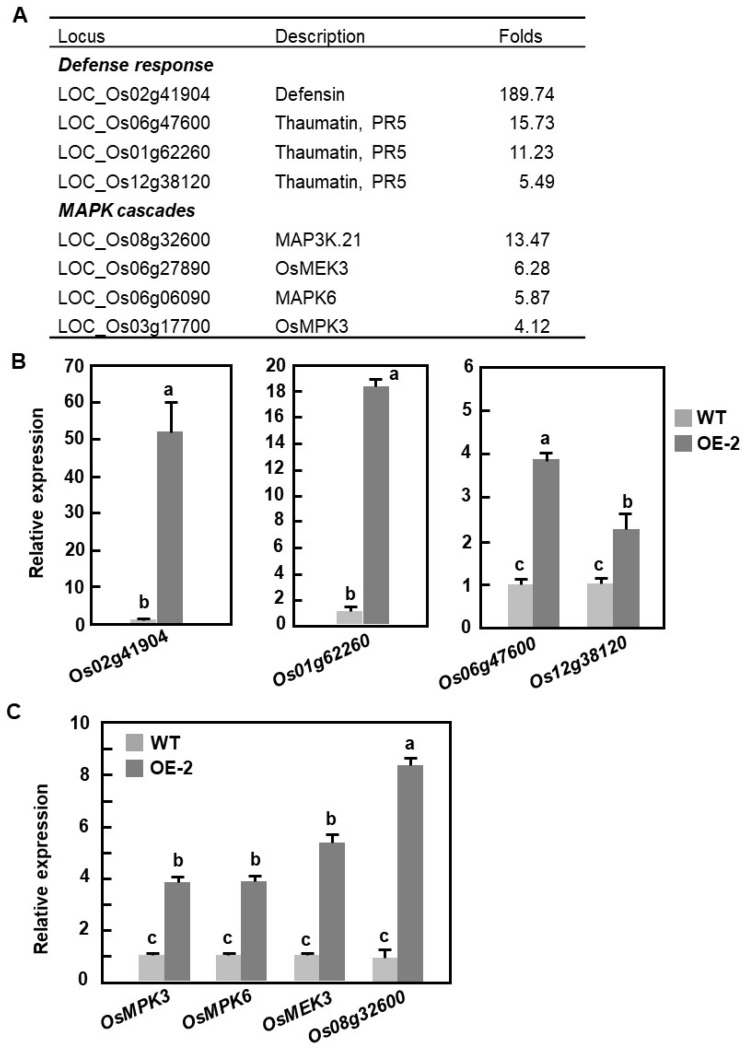
Up-regulated expression of defense and MAPK cascade genes in OsBIERF3-OE plants. (**A**) A selected list of up-regulated defense and MAPK cascade genes in OsBIERF3-OE plants as revealed by microarray analyses. (**B**,**C**) Up-regulated expression of defense (**B**) and MAPK cascade (**C**) genes in OsBIERF3-OE plants as validated by qRT-PCR analyses. Data presented in (**B**,**C**) are the means ± SD from three independent experiments and different letters indicate statistically significant difference at *p* < 0.05 level.

To gain further insights into the mechanism of OsBIERF3-regulated immunity against *M. oryzae* and *X. oryzae* pv. *oryzae*, gene expression profiles between 3-week-old OsBIERF3-OE and WT plants grown under normal conditions were determined and compared using the Affymetrix rice gene chip. A total of 3637 genes (2149 up-regulated and 1488 down-regulated) exhibited 2-fold (*p* < 0.05) changes in the transcript levels in OsBIERF3-OE-2 plants, compared with those in WT plants, and were identified as differentially expressed genes (Table S1). These up-regulated genes included 9 genes for PRs such as defensin, thaumatin, osmotin, and Bet VI family protein PR10, 7 genes encoding for components in MAPK cascades, 18 for LRR R-like proteins, 46 for receptor-like kinases and protein kinases, 31 for zinc finger proteins, 39 for transcription factors belonging to ERF, WRKY, bHLH, and MYB families, 7 for cytochrome P450, and 11 for cell wall synthetic enzymes (Table S1). qRT-PCR analyses verified the up-regulated expression of some of the selected differentially expressed genes in OsBIERF3-OE plants (Figure 5A,B,C). Notably, genes for OsMPK3, OsMPK6, and OsMEK3, well-known MAPK cascade components that play critical roles in rice immunity [96,97,98,99,100], were markedly up-regulated in OsBIERF3-OE plants, giving 2.95-, 3.11-, and 4.32-fold increases over those in WT plants (Figure 5C), implying the involvement of OsBIERF3 in the transcriptional regulation of the MAPK cascade. Collectively, these data suggest that overexpression of *OsBIERF3* in OsBIERF3-OE plants confers enhanced immunity against *M. oryzae* and *X. oryzae* pv. *oryzae* through the transcriptional regulation of MAPK cascades.

### 2.7. Altered Expression of OsBIERF3 Affected Cell Wall Thickness

**Figure 6:**
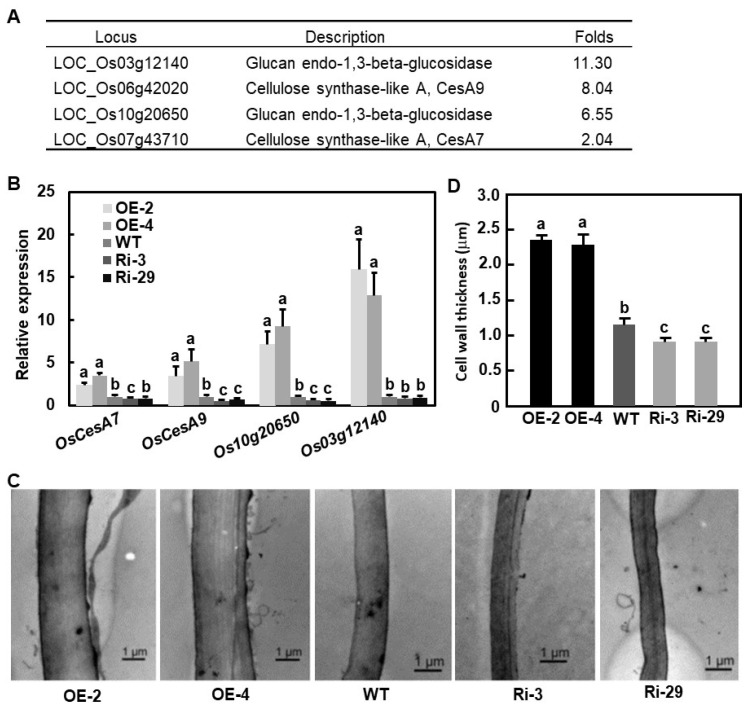
*OsBIERF3* affects cell wall thickness in rice. (**A**) Up-regulated expression of cell wall synthetic genes in OsBIERF3-OE plants as revealed by microarray analyses. (**B**) Up-regulation of cell wall synthetic genes in OsBIERF3-OE plants as validated by qRT-PCR analyses. (**C**) Representative TEM photographs showing cell walls in OsBIER3-OE and OsBIERF3-Ri plants. (**D**) Cell wall thickness. Sheaths from five individual plants were examined and at least 10 measurements were carried out for each of the sheath sections. Experiments in (**C**) were repeated for three times with similar results. Data presented in (**B**,**D**) are the means ± SD from three independent experiments and different letters indicate statistically significant difference at *p* < 0.05 level.

In the gene expression profiling data, a set of 11 genes encoding cell wall synthetic enzymes such as cellulose synthases and glucan endo-1,3-beta-glucosidases were significantly up-regulated in OsBIERF3-OE plants (Table S1). qRT-PCR analyses verified that the expression levels of two cellulose synthase genes, *OsCes7* and *OsCesA9,* and two glucan endo-1,3-beta-glucosidase genes, *Os10g20650* and *Os03g12140,* in OsBIERF3-OE plants were significantly up-regulated, as compared with those in WT plants (Figure 6A,B). By contrast, the expression levels of *OsCes9* and *Os10g20650* were down-regulated in OsBIERF3-Ri plants, in comparison to those in WT plants (Figure 6B). These results raised the possibility that OsBIERF3 is involved in cell wall formation. To test this hypothesis, we examined and measured the cell wall thickness in sheath tissues of OsBIERF3-OE, OsBIERF3-Ri, and WT plants under transmission electron microscopy (TEM). The cell walls in sheath tissues of OsBIERF3-OE plants became much thicker, leading to an increase of 1.1-fold, while the cell walls in sheath tissues of OsBIERF3-Ri plants were much thinner, resulting in a decrease of 21–24%, as compared with that in WT plants (Figure 6C,D). These data indicate that OsBIERF3 plays a role in modulating cell wall biosynthesis in rice.

### 2.8. OsBIERF3 Negatively Regulates Cold Tolerance but Does Not Affect Drought and Salt Tolerance

**Figure 7:**
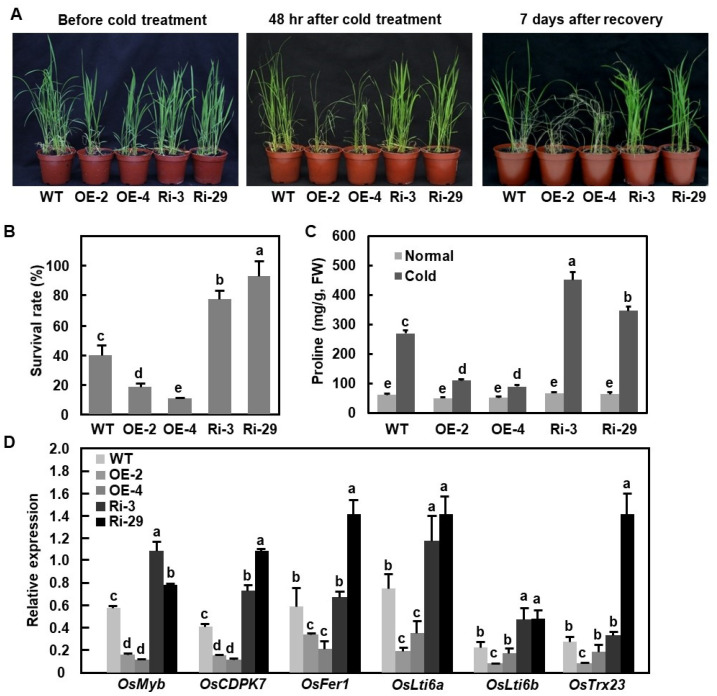
OsBIERF3 negatively regulates cold tolerance in rice. (**A**) Phenotype of OsBIERF3-OE, OsBIERF3-Ri, and WT plants before (left), at 48 h after cold treatment (middle), and at 7 days after recovery from cold treatment (right). (**B**) Survival rate of the cold-stressed OsBIERF3-OE, OsBIERF3-Ri, and WT plants at 9 days after recovery. (**C**) Proline contents in leaves of the cold-stressed OsBIERF3-OE, OsBIERF3-Ri, and WT plants at 2 days after cold treatment. (**D**) Expression of selected cold-tolerance-related genes in OsBIERF3-OE, OsBIERF3-Ri, and WT plants. Four-week-old plants were cold stressed by placing in a 4 °C growth chamber for 48 h and then recovered by moving to normal growth conditions for 7 days. Leaf samples were collected 2 days after cold treatment for measurement of proline contents. Experiments in (**A**) were repeated three times with similar results. Data presented in (**B**–**D**) are the mean ± SD from three independent experiments and different letters above the columns indicate statistically significant difference at *p* < 0.05 level.

The fact that the expression of *OsBIERF3* was up-regulated by drought, salt, and cold stress led us to examine whether OsBIERF3 plays role in abiotic stress tolerance by phenotyping OsBIERF3-OE and OsBIERF3-Ri plants under drought, salt, and cold stress conditions. In repeated drought stress experiments, drought symptoms, represented by rolled leaves and wilted plants, in OsBIERF3-OE and OsBIERF3-Ri plants at 10 days after drought treatment and at 7 days after re-watering was indistinguishable from WT plants (Figure S4A,B). Similarly, growth performance, root length, and shoot length of OsBIERF3-OE and OsBIERF3-Ri plants on 1/2 MS medium without NaCl supplement or with 150 mM NaCl were comparable to WT plants (Figure S4C–E). By contrast, OsBIERF3-OE plants displayed more severe while OsBIERF3-Ri plants exhibited milder cold damage symptoms, such as rolled leaves and wilted plants, as compared with WT plants, at 48 h after cold (4 °C) treatment and at 7 days after recovery (Figure 7A). At 7 days after recovery from cold stress, OsBIERF3-OE plants showed a lower survival rate (<20%) while OsBIERF3-Ri plants had a higher survival rate (>78%), as compared with that of WT plants (~40%) (Figure 6B). Free proline is an important compatible osmolyte that protects subcellular structures and macromolecules of plants under abiotic stress. To obtain further insights into the possible mechanism responsible for the involvement of OsBIERF3 in cold tolerance, the changes in proline content and the expression of several selected cold-responsive genes in OsBIERF3-OE and OsBIERF3-Ri plants were analyzed and compared with those in WT plants. Under normal conditions, proline contents in OsBIERF3-OE and OsBIERF3-Ri plants were comparable to that in WT plants (Figure 6C). Under cold stress, proline contents in OsBIERF3-OE plants significantly decreased, leading to a reduction of >61%, while the contents in OsBIERF3-Ri plants markedly increased, resulting in an increase of >23%, as compared with that in WT plants (Figure 6C). Similarly, the expression levels of cold-responsive genes *OsMyb*, *OsCDPK7*, *OsFer1*, *OsLti6a*, *OsLti6b*, and *OsTrx23* [101,102,103,104,105] in OsBIERF3-OE plants were markedly down-regulated, while the expression levels of these genes in OsBIERF3-Ri plants were significantly up-regulated, as compared with those in WT plants (Figure 6D). Together, these results indicate that OsBIERF3 negatively regulates cold tolerance but is not involved in drought and salt tolerance in rice.

### 2.9. Involvement of ET and ABA in OsBIERF3-Mediated Cold Response in Rice

**Figure 8:**
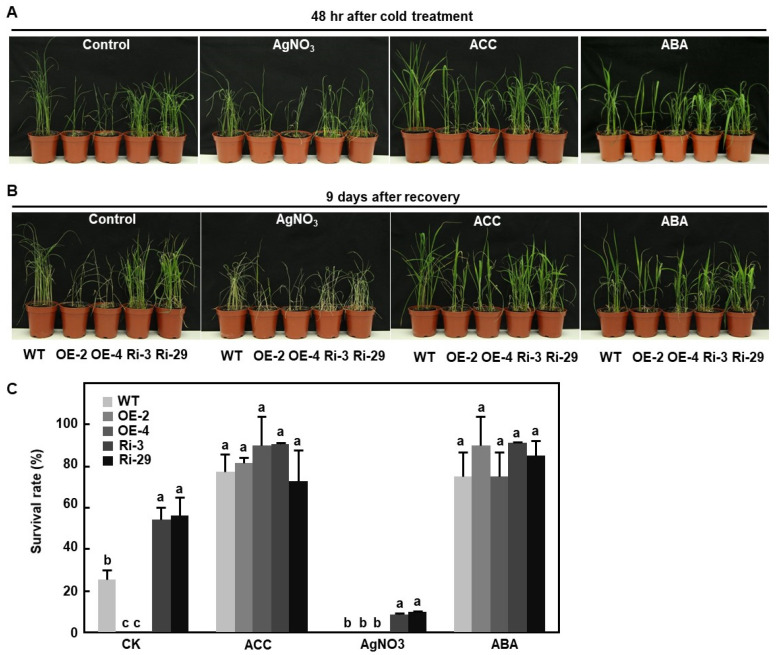
Function of OsBIERF3 in cold tolerance depends on ABA and ET pathways. (**A**) Phenotype of cold damage in OsBIERF3-OE, OsBIERF3-Ri, and WT plants at 48 h after cold treatment. (**B**) Growth phenotype of the cold-stressed OsBIERF3-OE, OsBIERF3-Ri, and WT plants at 9 days after recovery from cold treatment. (**C**) Survival rate of the cold stressed OsBIERF3-OE, OsBIERF3-Ri, and WT plants at 9 days after recovery. Four-week-old plants were treated by foliar spraying with 100 μM ACC, 100 μM AgNO_3_, 100 μM ABA or similar volume of distilled sterilized water and then subjected to cold treatment by placing in a 4 °C freezer for 48 h, followed by recovery to normal growth condition. Experiments in (**A**,**B**) were repeated three times with similar results. Data presented in (**C**) are the mean ± SD from three independent experiments and different letters above the columns indicate statistically significant difference at *p* < 0.05 level.

ABA and ET are key regulators of signaling pathways during the plant response to abiotic stress, including cold [28,106,107,108]. The responsiveness of *OsBIERF3* to ET and ABA (Figure 1B,C) led us to examine whether ET and ABA are involved in *OsBIERF3*-mediated cold response in rice. To test this possibility, we analyzed the effect of pretreatment with ACC, AgNO_3_ (an inhibitor of ET action) [109], and ABA on cold tolerance in OsBIERF3-OE and OsBIERF3-Ri plants. At 48 h after cold stress, the ACC- or ABA-treated rice plants of all tested genotypes showed milder damage, while the pretreated AgNO_3_-rice plants displayed more severe cold damage phenotype, e.g., rolled leaves and wilted plants, as compared with those of rice plants without pretreatment (Figure 8A), especially for the ACC- or ABA-treated OsBIERF3-OE plants. At 9 days after recovery from cold stress, the ACC- or ABA-treated rice plants grew better (Figure 8B) and the survival rates for all tested genotypes were significantly higher than those of rice plants without pretreatment (Figure 8C). OsBIERF3-OE plants showed a particularly similar survival rate to those of OsBIERF3-Ri and WT plants (Figure 8C). By contrast, the AgNO_3_-treated rice plants exhibited more severe cold damage than rice plants without pretreatment (Figure 8B). None of the AgNO_3_-treated OsBIERF3-OE and WT plants survived, and the survival rate of the AgNO_3_-treated OsBIERF3-Ri plants was also significantly decreased, as compared with the rice plants without pretreatment, at 9 days after recovery from cold treatment (Figure 8C). These results indicate that ET and ABA are not only essential for cold stress response but also required for OsBIERF3-mediated cold response in rice.

## 3. Discussion

OsBIERF3 (OsERF#091) belongs to group IX of the ERF subfamily [30]. Arabidopsis ERFs from group IX of the ERF subfamily play critical roles in immunity [110,111] and abiotic stress response [28]. There are 19 members in group IX of the rice ERF subfamily [30,31], and four of them, OsBIERF3, OsERF083, OsERF123, and OsERF922 (OsERF092), have been shown to be involved in immunity [48,50,51,58]. OsBIERF3 and OsERF083 positively regulate immunity [50,51], while OsERF092 and OsERF123 act as a negative regulator of immunity against *M. oryzae* or as a susceptibility gene for *X. oryzae* pv. *oryzae* [48,58]. We previously showed that the overexpression of *OsBIERF3* in tobacco conferred an increased resistance against viral and bacterial pathogens [51]. The present study further demonstrated using OsBIERF3-OE and OsBIERF3-Ri transgenic rice lines that OsBIERF3 positively regulates immunity against *M. oryzae* and *X. oryzae* pv. *oryzae*, but negatively regulates cold tolerance in rice.

Expression of *OsBIERF3*, *OsERF083*, and *OsERF092* was induced by *M. oryzae* [46,48,50]. The present study revealed that the expression of *OsBIERF3* was induced by *X. oryzae* pv. *oryzae* and the induction was much stronger and earlier in the incompatible interaction between rice and *X. oryzae* pv. *oryzae*, but was weaker and slower during the compatible interaction (Figure 1A). This is similar to the expression induction of *OsBIERF3* and *OsERF092* in the incompatible and compatible interactions between rice and *M. oryzae* [46,48]. Furthermore, the expression of *OsBIERF3* was induced by an SA analog benzothiadiazole [46], and by SA, MeJA, and ACC (Figure 1B), which is similar to *OsERF083* and *OsERF087*, which were induced by SA, JA, and ethephon [50,112]. Phytohormones such as SA, JA, and ET play critical roles in fine-tuning immunity in rice [113,114,115]. The induction of *OsBIERF3* expression by pathogens and defense signaling hormones implies the involvement of *OsBIERF3* in rice immunity.

Biochemically, OsBIERF3, OsERF083, OsERF087, and OsERF092, were previously shown to bind to a canonical GCC box-containing sequence [46,48,50,112]. The present study further revealed that OsBIERF3 had transcriptional activation activity in yeast and this activity depended on the C-terminal region (Figure 2), demonstrating that OsBIERF3 is a transcriptional activator. This is similar to OsERF092, OsERF87, and OsERF136, which showed transcriptional activator activity in rice cells through binding to GCC box elements [48,112]. It is therefore reasonable to speculate that OsBIERF3 plays its role in immunity through activating downstream target genes that are involved in defense response and immune signaling. In fact, microarray-based expression profiling analyses revealed that more than 2100 genes were up-regulated in OsBIERF3-OE plants (Supplementary Table S1), and a set of genes encoding defensive proteins such as defensin, thaumatin, osmotin, and Bet VI family protein was identified (Figure 5A, Supplementary Table S1). Importantly, the expression levels of *OsMPK3*, *OsMPK6*, and *OsMEK3*, which are involved in rice immunity against *M. oryzae*, *X. oryzae* pv. *oryzae*, and chewing herbivore insects [96,97,98,99,100], were up-regulated in OsBIERF3-OE plants (Figure 6B,C), implying the involvement of OsBIERF3 in the transcriptional regulation of these well-known immunity-related MAPK cascade components. This is similar to OsERF3, an EAR-motif-containing ERF that is involved in resistance to herbivore insects, which positively affected the transcript levels of two MAPK genes [59]. Further bioinformatics analyses revealed the presence of GCC box elements in the promoter regions of some of the genes, including *OsMPK3* and *OsPR10* (LOC_Os04g39150), that were up-regulated in OsBIERF3-OE plants. Recently, it was found that OsERF87 and OsERF136, which belong to a different clade of group IX ERFs, directly bind to the promoter region of *RSOsPR10*, a root-specific *OsPR10* gene, and activate its expression [112]. It is thus likely that some of the up-regulated genes in OsBIERF3-OE plants may be putative OsBIERF3 targets, which need to be further examined.

Studies have demonstrated that plant cell walls act as structural barriers to prevent pathogen penetration and colonization and thus play important roles in immune responses against diverse pathogens [116,117,118]. In rice, it has been shown that cell wall structure and integrity are critical to immunity against *M. oryzae* and *X. oryzae* pv. *oryzae* [119,120,121]. In the present study, we observed that OsBIERF3-OE plants develop thicker cell walls while OsBIERF3-Ri plants generate thinner walls compared with corresponding WT plants (Figure 6C,D), accompanied by the up-regulated expression of cell wall biosynthetic genes, as revealed by microarray and qRT-PCR analyses (Figure 6A,B). Bioinformatics analyses indicated that the promoter of *OsCesA9*, encoding a catalytic subunit of the cellulose synthase complex that is responsible for cellulose synthesis on the secondary cell wall [122,123], contains typical GCC box elements, implying that OsBIERF3 may directly regulate the expression of *OsCesA9* and thus affect the formation of the cell wall in rice. Collectively, these observations suggest that OsBIERF3 functions in rice immunity against *M. oryzae* and *X. oryzae* pv. *oryzae*, probably through regulating the cell wall synthesis pathway.

The expression of *OsBIERF3* was induced by drought, salt, and cold stress [46], as well as by stress hormones ABA and ET (Figure 1B–E), implying the involvement of *OsBIERF3* in the abiotic stress response in rice. Surprisingly, OsBIERF3-OE and OsBIERF3-Ri plants did not show any alteration in drought and salt tolerance (Figure S4), indicating that OsBIERF3 is not involved in drought and salt stress response. By contrast, OsBIERF3-OE plants attenuated while OsBIERF3-Ri plants increased cold tolerance (Figure 7A,B), accompanied by the altered accumulation of proline and the expression of cold-responsive genes (Figure 7C,D), revealing that OsBIERF3 is a negative regulator of rice cold tolerance. Because OsBIERF3 is a transcriptional activator (Figure 2), it is thus likely that OsBIERF3 activates some unknown negative regulators that repress the cold stress response, instead of directly suppressing cold-stress-responsive genes. This is similar to OsERF092, which is a transcriptional activator and negatively regulates salt tolerance in rice [48]. ABA and ET play critical roles in signaling pathways of plant response to diverse abiotic stresses [28,106,107,108]. Blocking ABA biosynthesis through knockout of 9-*cis*-epoxycarotenoid dioxygenase genes significantly decreased abiotic stress tolerance in rice [124,125]. Exogenous ABA restored the attenuated cold tolerance in OsBIERF3-OE plants (Figure 8), implying the involvement of ABA involved in OsBIERF3-mediated cold stress response. On the other hand, exogenous ACC, a precursor of ET, restored the attenuated cold tolerance in OsBIERF3-OE plants, while pretreatment of OsBIERF3-Ri plants with AgNO_3_, an inhibitor of ET action [109], significantly suppressed the enhanced cold tolerance in OsBIERF3-Ri plants (Figure 8), indicating that ET is involved in the OsBIERF3-mediated rice cold stress response. This is similar to the previous observations that OsERF109 and OsERF3 function in abiotic stress response via affecting ET biosynthesis [70,80,81]. Taken together, it is likely that ABA and ET are required for the function of OsBIERF3 in the rice cold stress response.

In summary, our functional analyses using overexpression and RNAi-mediated suppression transgenic rice lines demonstrate that OsBIERF3, as a transcriptional activator, positively contributes to resistance against *M. oryzae* and *X. oryzae* pv. *oryzae* but negatively regulates cold tolerance in rice. Manipulation of *OsBIERF3* in rice had no deleterious impact on agronomic traits such as plant growth/development and grain yield as well as drought and salt stress tolerance. OsBIERF3 may offer promising potential for application of *OsBIERF3* to develop novel disease-resistant rice materials/varieties that can be used in temperate regions where cold stress is not the case. Further global mapping of the genome-wide DNA-binding sites and characterization of the direct target genes of OsBIERF3 will provide deeper insights into the molecular basis of OsBIERF3-mediated broad-spectrum immunity and cold stress response in rice.

## 4. Materials and Methods

### 4.1. Plant Growth and Treatments

Rice cv. Yuanfengzao was used for the analysis of gene expression by hormone and abiotic stress treatments while a pair of near-isogenic lines, IR24 and BB10 [93,94], was used for analysis of gene expression in rice–*X. oryzae* pv. *oryzae* interactions. Rice plants were grown in a growth room under 28 °C 14 h light/26 °C 10 h dark cycle, and 80–85% relative humidity. For bacterial inoculation, rice plants of varieties IR24 and BB10 at the booting stage were inoculated with *X. oryzae pv. oryzae* strain PXO86 using the leaf-clipping method [126]. For hormone treatment, 2-week-old plants were sprayed with 100 μM MeJA, 100 μM ACC, 150 μM SA, and 100 μM ABA (Sigma-Aldrich, St. Louis, MO, USA) in a solution containing 0.1% ethanol and 0.02% Tween-20 or with the same volume of the solution as a mock control. For drought treatment, plants were placed on lab benches without water supply or on water-saturated filter papers as controls in Petri dishes [127]. For salt treatment, plants were irrigated with 150 mM NaCl or a similar volume of sterilized distilled water as controls [127]. For cold treatment, plants were transferred to a growth chamber with temperature set at 4 °C [128]. Leaf samples were collected at indicated time points, frozen in liquid nitrogen, and stored at −80 °C until use. Each treatment in each of the experiments included three biological replicates with at least three plants, and the experiments were independently repeated three times.

### 4.2. Generation and Characterization of OsBIERF3-OE and OsBIERF3-Ri Lines

For the construction of overexpression vector, the 912 bp coding sequence of OsBIERF3 was inserted into the pCoUm vector under the control of a maize Ubi promoter to generate pCoU-Ubi::OsBIERF3. For the construction of Ri vector, a 400 bp 5′-end fragment was used to construct a self-complementary hairpin vector pCoU-Ubi::OsBIERF3-Ri [129]. The resulting constructs were introduced into calli of rice cv. Xiushui 11 by the *Agrobacterium*-mediated transformation method. T2 generation of the obtained OsBIERF3-OE and OsBIERF3-Ri lines was screened by planting seeds on 1/2 MS medium supplemented with 50 μg/mL hygromycin (Hgr) and lines showing 3:1 (Hgr-resistant:Hgr-susceptible) segregation were selected as putative transgenic lines with a single copy of the transgene. Screening for homozygous lines and analysis of copy number of the transgene by Southern blotting assays was carried out as described previously [130]. Assessment of agronomic traits of OsBIERF3-OE and OsBIERF3-Ri lines was carried out as previously described [130].

### 4.3. Transcriptional Activation Assays

For transactivation assay, the coding sequence of OsBIERF3 was fused in-frame to the yeast GAL4 DNA binding domain in the vector pBD-GAL4Cam (Clontech, Mountain View, CA, USA) to produce pBD-OsBIERF3. The pBD-OsBIERF3 and pBD empty vector (negative control) were transformed into yeast strain AH109. The transformed yeasts were plated on SD/Trp- medium or SD/Trp-His- medium and incubated for 3 days at 30 °C, followed by the addition of X-α-gal. The transactivation activities of the fusion proteins were evaluated according to the growth situation and production of blue pigments after the addition of X-α-gal on the SD/Trp-His-medium. The experiments were independently repeated three times.

### 4.4. Disease Assays

For evaluation of blast resistance, 4-week-old seedlings were inoculated by foliar spraying with spore suspension (1 × 10^5^ spores/mL) of *M. oryzae* race ZE3 strain 97-220 [131]. The inoculated plants were kept in the dark for 24 h at 25 °C with 100% relative humidity and then moved to a normal growth environment. Disease phenotype was examined and numbers of lesions were counted from at least 30 leaves of 15 individual inoculated plants at 6 dpi. Dead cells in inoculated leaves were detected using the trypan blue staining method as previously described [130]. Relative fungal growth in inoculated rice leaves was measured using qRT-PCR [132] by analyzing and comparing the genomic level of *M. oryzae* 28S rDNA gene with that of the rice *eEF-1α* gene as an internal control. Dead cell staining and fungal growth were performed with 6 inoculated leaves of three individual plants. For evaluation of bacterial blight resistance, greenhouse-grown rice plants at the booting stage were inoculated with *X. oryzae* pv. *oryzae* strain PXO86 using the leaf-clipping method [126] and inoculated plants were kept in a greenhouse under environmental conditions at 30 °C in day/25 °C in night with natural sunlight. Disease phenotype was photographed and lesion length was measured from at least 30 leaves of 15 individual inoculated plants at 15 dpi. *X. oryzae* pv. *oryzae* growth in inoculated leaves was measured from six leaves of three individual plants by counting colony-forming units (CFU) on NA plates [133]. Each treatment in each of the experiments included three biological replicates with at least 10 plants, and the experiments were independently repeated three times.

### 4.5. Abiotic Stress Tolerance Assays

Abiotic stress tolerance assays were performed as described previously [127,128]. For drought tolerance assay, 4-week-old OsBIERF3-OE or OsBIERF3-Ri plants were grown with WT plants in the same barrels and were subjected to drought stress by stopping watering for 15 days, followed by re-watering for another 12 days. Plants with green leaves and healthy young leaves after re-watering were considered as survivals, and surviving plants were evaluated at 12 days after re-watering. For salt tolerance assay, 100 seeds were germinated on 1/2 MS medium supplemented with or without 150 mM NaCl under 28 °C/25 °C (day/night) with a 12 h photoperiod. At 6 days after germination, root length and shoot height of at least 30 plants were measured. For cold stress tolerance assay, 4-week-old OsBIERF3-OE or OsBIERF3-Ri plants were grown with WT plants in the same barrel and then transferred into a growth chamber with the temperature set at 4 °C with a cycle of 16 h light/8 h dark for 2 days, followed by transferring to the growth room with the normal condition for recovery. Plants with green leaves and healthy young leaves after transferring to the normal growth condition were considered as survivals, and surviving plants were evaluated at 7 days after recovery from cold treatment. Survival rate was calculated as the ratio of the number of survived plants over the total number of treated plants. Free proline content was determined using the colorimetric method [134] with a 0.5 g leaf sample. Each treatment in each of the experiments included three biological replicates with at least 10 plants, and the experiments were independently repeated three times.

### 4.6. Observation and Measurement of Cell Wall Thickness by TEM

Microscopic examination of cell wall thickness was carried out as described previously [135]. Briefly, sheath segments were collected from eight-week-old rice plants and fixed in 3% glutaraldehyde in phosphate buffer (100 mM, pH7.0) for at least 4 h, washed three times with the same phosphate buffer for 15 min each, and then post fixed in 1% osmium tetroxide in the phosphate buffer for 2 h. After washing three times, the sheath segments were embedded in Epon 812, and ultra-thin sections were stained by uranyl acetate and alkaline lead citrate for 20 min, respectively. Observation of the cell walls was performed under TEM of Model H-7650 (Hitachi, Tokyo, Japan). At least 10 ultra-thin sections were examined for each of the segment samples and the experiments were independently repeated three times with a minimum of 15 individual plants.

### 4.7. Microarray Analyses of Differentially Expressed Genes in OsBIERF3-OE Plants

Leaf samples were collected from 3-week-old OsBIERF3-OE and WT plants and total RNA was extracted using TRIzol reagent (Invitrogen, Shanghai, China). Two micrograms of total RNA were used for the synthesis of double-stranded cDNA, and biotin-tagged cRNA was prepared using a MessageAmp II cRNA Amplification Kit (Ambion, Foster City, CA, USA) according to the manufacturer’s instructions. The resulting biotin-tagged cRNA was fragmented to strands of 35–200 bases in length according to Affymetrix’s protocols. The fragmented cRNA was hybridized to Affymetrix Rice Genome Array containing 51,279 transcripts representing rice subspecies *japonica* and *indica* by standard Affymetrix protocol (CapitalBio Technology Company, Beijing, China). All procedures for probe preparation, hybridization, scanning, data collection, and bioinformatics analyses were carried out at the Beijing CapitalBio Technology Company (Beijing, China). Normalization was performed according to the standard Affymetrix protocols to allow the comparison of the samples and genes with a 2-fold change in the transcript level between OsBIERF3-OE and WT plants were defined as differentially expressed genes. Two independent biological samples for OsBIERF3-OE and WT plant were performed for microarray analyses and the differentially expressed genes with *p* < 0.05 were chosen.

### 4.8. qRT-PCR Analyses of Gene Expression

Total RNA was extracted from frozen leaf tissues using TRIzol (Invitrogen, Shanghai, China) and then treated with RNase-free DNase (TaKaRa, Dalian, China). First-strand cDNA was synthesized from 1 μg total RNA using AMV reverse transcriptase (TaKaRa, Dalian, China) according to the manufacturer’s recommendations. Each qPCR reaction contained 12.5 μL 2 × Fast essential (Roche Diagnostics, Shanghai, China), 1 μg cDNA and 10 μmol of each gene-specific primer in a final volume of 25 μL. The qPCR was performed on a CFX96 real-time PCR detection system (BioRad, Hercules, CA, USA). Data obtained were normalized using rice *OsActin* as an internal control and relative expression level was calculated using the 2^-ΔΔCT^ method. Primer information is provided in Table S2. Each treatment in each of the experiments included three biological replicates with at least three plants, and the experiments were independently repeated three times.

## Data Availability

All data generated by this study are available upon request.

## Supplementary Materials

The following are available online at https://www.mdpi.com/article/10.3390/ijms23020606/s1.

## References

[1] Nakashima K., Yamaguchi-Shinozaki K., Shinozaki K. (2014). The transcriptional regulatory network in the drought response and its crosstalk in abiotic stress responses including drought, cold, and heat. Front. Plant Sci..

[2] Takahashi F., Kuromori T., Urano K., Yamaguchi-Shinozaki K., Shinozaki K. (2020). Drought stress responses and resistance in plants: From cellular responses to long-distance intercellular communication. Front. Plant Sci..

[3] Yu Z., Duan X., Luo L., Dai S., Ding Z., Xia G. (2020). How plant hormones mediate salt stress responses. Trends Plant Sci..

[4] Lamers J., van der Meer T., Testerink C. (2020). How plants sense and respond to stressful environments. Plant Physiol..

[5] Zhang H., Zhao Y., Zhu J.K. (2020). Thriving under stress: How plants balance growth and the stress response. Dev. Cell.

[6] Zhou J.M., Zhang Y. (2020). Plant immunity: Danger perception and signaling. Cell.

[7] Ku Y.S., Sintaha M., Cheung M.Y., Lam H.M. (2018). Plant hormone signaling crosstalks between biotic and abiotic stress responses. Int. J. Mol. Sci..

[8] Bürger M., Chory J. (2019). Stressed out about hormones: How plants orchestrate immunity. Cell Host Microbe.

[9] Li N., Han X., Feng D., Yuan D., Huang L.J. (2019). Signaling crosstalk between salicylic acid and ethylene/jasmonate in plant defense: Do we understand what they are whispering?. Int. J. Mol. Sci..

[10] Zandalinas S.I., Fritschi F.B., Mittler R. (2020). Signal transduction networks during stress combination. J. Exp. Bot..

[11] Saijo Y., Loo E.P. (2020). Plant immunity in signal integration between biotic and abiotic stress responses. New Phytol..

[12] Tsuda K., Somssich I.E. (2015). Transcriptional networks in plant immunity. New Phytol..

[13] Li B., Meng X., Shan L., He P. (2016). Transcriptional regulation of pattern-triggered immunity in plants. Cell Host Microbe.

[14] Kim J.H. (2021). Multifaceted chromatin structure and transcription changes in plant stress response. Int. J. Mol. Sci..

[15] Chen J., Clinton M., Qi G., Wang D., Liu F., Fu Z.Q. (2020). Reprogramming and remodeling: Transcriptional and epigenetic regulation of salicylic acid-mediated plant defense. J. Exp. Bot..

[16] Buscaill P., Rivas S. (2014). Transcriptional control of plant defence responses. Curr. Opin. Plant Biol..

[17] Birkenbihl R.P., Liu S., Somssich I.E. (2017). Transcriptional events defining plant immune responses. Curr. Opin. Plant Biol..

[18] Amorim L.L.B., da Fonseca Dos Santos R., Neto J.P.B., Guida-Santos M., Crovella S., Benko-Iseppon A.M. (2017). Transcription factors involved in plant resistance to pathogens. Curr. Protein Pept. Sci..

[19] Ng D.W., Abeysinghe J.K., Kamali M. (2018). Regulating the regulators: The control of transcription factors in plant defense signaling. Int. J. Mol. Sci..

[20] Ashapkin V.V., Kutueva L.I., Aleksandrushkina N.I., Vanyushin B.F. (2020). Epigenetic mechanisms of plant adaptation to biotic and abiotic stresses. Int. J. Mol. Sci..

[21] Nuruzzaman M., Sharoni A.M., Kikuchi S. (2013). Roles of NAC transcription factors in the regulation of biotic and abiotic stress responses in plants. Front. Microbiol..

[22] Huang P.Y., Catinot J., Zimmerli L. (2016). Ethylene response factors in Arabidopsis immunity. J. Exp. Bot..

[23] Phukan U.J., Jeena G.S., Shukla R.K. (2016). WRKY transcription factors: Molecular regulation and stress responses in plants. Front. Plant Sci..

[24] Zanetti M.E., Rípodas C., Niebel A. (2017). Plant NF-Y transcription factors: Key players in plant-microbe interactions, root devel-opment and adaptation to stress. Biochim. Biophys. Acta.

[25] Noman A., Liu Z., Aqeel M., Zainab M., Khan M.I., Hussain A., Ashraf M.F., Li X., Weng Y., He S. (2017). Basic leucine zipper domain transcription factors: The vanguards in plant immunity. Biotechnol. Lett..

[26] Wang X., Niu Y., Zheng Y. (2021). Multiple functions of MYB transcription factors in abiotic stress responses. Int. J. Mol. Sci..

[27] Feng K., Hou X.L., Xing G.M., Liu J.X., Duan A.Q., Xu Z.S., Li M.-Y., Zhuang J., Xiong A.-S. (2020). Advances in AP2/ERF super-family transcription factors in plant. Crit. Rev. Biotechnol..

[28] Xie Z., Nolan T.M., Jiang H., Yin Y. (2019). AP2/ERF transcription factor regulatory networks in hormone and abiotic stress responses in Arabidopsis. Front. Plant Sci..

[29] Wani S.H., Anand S., Singh B., Bohra A., Joshi R. (2021). WRKY transcription factors and plant defense responses: Latest discoveries and future prospects. Plant Cell Rep..

[30] Nakano T., Suzuki K., Fujimura T., Shinshi H. (2006). Genome-wide analysis of the ERF gene family in Arabidopsis and rice. Plant Physiol..

[31] Sharoni A.M., Nuruzaman M., Satoh K., Shimizu T., Kondoh H., Sasaya T., Choi R., Omura T., Kikuchi S. (2011). Gene structures, classification and expression models of the AP2/EREBP transcription factor family in rice. Plant Cell Physiol..

[32] Ohme-Takagi M., Shinshi H. (1995). Ethylene-inducible DNA binding proteins that interact with an ethylene-responsive element. Plant Cell.

[33] Hao D., Ohme-Takagi M., Sarai A. (1998). Unique mode of GCC box recognition by the DNA-binding domain of ethylene-responsive element binding factor (ERF domain) in plant. J. Biol. Chem..

[34] Hao D., Yamasaki K., Sarai A., Ohme-Takagi M. (2002). Determinants in the sequence specific binding of two plant transcription factors, CBF1 and NtERF2, to the DRE and GCC motifs. Biochemistry.

[35] Brown R.L., Kazan K., McGrath K.C., Maclean D.J., Manners J.M. (2003). A role for the GCC-box in jasmonate-mediated activation of the *PDF1.2* gene of Arabidopsis. Plant Physiol..

[36] Van der Does D., Leon-Reyes A., Koornneef A., Van Verk M.C., Rodenburg N., Pauwels L., Goossens A., Körbes A.P., Memelink J., Ritsema T. (2013). Salicylic acid suppresses jasmonic acid signaling downstream of SCFCOI1-JAZ by targeting GCC promoter motifs via transcription factor ORA59. Plant Cell.

[37] Fujimoto S.Y., Ohta M., Usui A., Shinshi H., Ohme-Takagi M. (2000). Arabidopsis ethylene-responsive element binding factors act as transcriptional activators or repressors of GCC box-mediated gene expression. Plant Cell.

[38] Licausi F., Ohme-Takagi M., Perata P. (2013). APETALA2/Ethylene Responsive Factor (AP2/ERF) transcription factors: Mediators of stress responses and developmental programs. New Phytol..

[39] Müller M., Munné-Bosch S. (2015). Ethylene response factors: A key regulatory hub in hormone and stress signaling. Plant Physiol..

[40] Dey S., Vlot A.C. (2015). Ethylene responsive factors in the orchestration of stress responses in monocotyledonous plants. Front. Plant Sci..

[41] Giuntoli B., Perata P. (2018). Group VII ethylene response factors in Arabidopsis: Regulation and physiological roles. Plant Physiol..

[42] Shoji T., Yuan L. (2021). ERF gene clusters: Working together to regulate metabolism. Trends Plant Sci..

[43] Xu Z.S., Chen M., Li L.C., Ma Y.Z. (2011). Functions and application of the AP2/ERF transcription factor family in crop improvement. J. Integr. Plant Biol..

[44] Srivastava R., Kumar R. (2018). The expanding roles of APETALA2/Ethylene Responsive Factors and their potential applications in crop improvement. Br. Funct. Genom..

[45] Debbarma J., Sarki Y.N., Saikia B., Boruah H.P.D., Singha D.L., Chikkaputtaiah C. (2019). Ethylene response factor (ERF) family proteins in abiotic stresses and CRISPR-Cas9 genome editing of ERFs for multiple abiotic stress tolerance in crop plants: A review. Mol. Biotechnol..

[46] Cao Y., Song F., Goodman R., Zheng Z. (2006). Molecular characterization of four rice ethylene-responsive element binding protein genes and their expressions in response to biotic and abiotic stresses. J. Plant Physiol..

[47] Lin R., Zhao W., Meng X., Peng Y. (2007). Molecular cloning and characterization of a rice gene encoding AP2/EREBP-type transcription factor and its expression in response to infection with blast fungus and abiotic stresses. Physiol. Mol. Plant Pathol..

[48] Liu D.F., Chen X.J., Liu J.Q., Ye J.C., Guo Z.J. (2012). The rice ERF transcription factor OsERF922 negatively regulates resistance to *Magnaporthe oryzae* and salt tolerance. J. Exp. Bot..

[49] Kim C.Y., Lee S., Park H.C., Chang C.G., Cheong Y.H., Choi Y.J., Han C., Lee S.Y., Lim C.O., Cho M.J. (2000). Identification of rice blast fungal elicitor-responsive genes by differential display analysis. Mol. Plant-Microbe Interact..

[50] Tezuka D., Kawamata A., Kato H., Saburi W., Mori H., Imai R. (2019). The rice ethylene response factor OsERF83 positively regulates disease resistance to *Magnaporthe oryzae*. Plant Physiol. Biochem..

[51] Cao Y.F., Wu Y., Zheng Z., Song F.M. (2006). Overexpression of the rice EREBP-like gene *OsBIERF3* enhances disease resistance and salt tolerance in transgenic tobacco. Physiol. Mol. Plant Pathol..

[52] Pillai S.E., Kumar C., Dasgupta M., Kumar B.K., Vungarala S., Patel H.K., Sonti R.V. (2020). Ectopic expression of a cell-wall-degrading enzyme-induced *OsAP2/ERF152* leads to resistance against bacterial and fungal infection in Arabidopsis. Phytopathology.

[53] Wang F.J., Wang C.L., Liu P.Q., Lei C.L., Hao W., Liu Y., Zhao K. (2016). Enhanced rice blast resistance by CRISPR/Cas9-tagarted mutagenesis of the ERF transcription factor gene *OsERF922*. PLoS ONE.

[54] Sahu A., Das A., Saikia K., Barah P. (2020). Temperature differentially modulates the transcriptome response in *Oryza sativa* to *Xanthomonas oryzae* pv. *oryzae* infection. Genomics.

[55] Cheong Y.H., Moon B.C., Kim J.K., Kim C.Y., Kim M.C., Kim I.H., Park C.Y., Park B.O., Koo S.C., Yoon H.W. (2003). BWMK1, a rice mitogen-activated protein kinase, locates in the nucleus and mediates pathogenesis-related gene expression by activation of a transcription factor. Plant Physiol..

[56] Seo Y.S., Chern M., Bartley L.E., Han M., Jung K.H., Lee I., Walia H., Richter T., Xu X., Cao P. (2011). Towards establishment of a rice stress response interactome. PLoS Genet..

[57] Jisha V., Dampanaboina L., Vadassery J., Mithofer A., Kappara S., Ramanan R. (2015). Overexpression of an AP2/ERF type transcription factor *OsEREBP1* confers biotic and abiotic stress tolerance in rice. PLoS ONE.

[58] Tran T.T., Pérez-Quintero A.L., Wonni I., Carpenter S.C.D., Yu Y., Wang L., Leach J.E., Verdier V., Cunnac S., Bogdanove A.J. (2018). Functional analysis of African *Xanthomonas oryzae* pv. *oryzae* TALomes reveals a new susceptibility gene in bacterial leaf blight of rice. PLoS Pathog..

[59] Lu J., Ju H., Zhou G., Zhu C., Erb M., Wang X., Wang P., Lou Y. (2011). An EAR-motif-containing ERF transcription factor affects herbivore-induced signaling, defense and resistance in rice. Plant J..

[60] Xu K., Xu X., Fukao T., Canlas P., Maghirang-Rodriguez R., Heuer S., Ismail A.M., Bailey-Serres J., Ronald P.C., Mackill D.J. (2006). Sub1A is an ethylene-response-factor-like gene that confers submergence tolerance to rice. Nature.

[61] Fukao T., Xu K., Ronald P.C., Bailey-Serres J. (2006). A variable cluster of ethylene response factor-like genes regulates metabolic and developmental acclimation responses to submergence in rice. Plant Cell.

[62] Hattori Y., Nagai K., Furukawa S., Song X.-J., Kawano R., Sakakibara H., Wu J., Matsumoto T., Yoshimura A., Kitano H. (2009). The ethylene response factors SNORKEL1 and SNORKEL2 allow rice to adapt to deep water. Nature.

[63] Schmitz A.J., Folsom J.J., Jikamaru Y., Ronald P., Walia H. (2013). SUB1A-mediated submergence tolerance response in rice involves differential regulation of the brassinosteroid pathway. New Phytol..

[64] Dubouzet J.G., Sakuma Y., Ito Y., Kasuga M., Dubouzet E.G., Miura S., Seki M., Shinozaki K., Yamaguchi-Shinozaki K. (2003). *OsDREB* genes in rice, *Oryza sativa* L., encode transcription activators that function in drought-, high-salt- and cold-responsive gene expression. Plant J..

[65] Chen J.Q., Meng X.P., Zhang Y., Xia M., Wang X.P. (2008). Over-expression of *OsDREB* genes lead to enhanced drought tolerance in rice. Biotechnol. Lett..

[66] Wang Q., Guan Y., Wu Y., Chen H., Chen F., Chu C. (2008). Overexpression of a rice *OsDREB1F* gene increases salt, drought, and low temperature tolerance in both Arabidopsis and rice. Plant Mol. Biol..

[67] Cui M., Zhang W., Zhang Q., Xu Z., Zhu Z., Duan F., Wu R. (2011). Induced over-expression of the transcription factor *OsDREB2A* improves drought tolerance in rice. Plant Physiol. Biochem..

[68] Mallikarjuna G., Mallikarjuna K., Reddy M.K., Kaul T. (2011). Expression of *OsDREB2A* transcription factor confers enhanced dehydration and salt stress tolerance in rice (*Oryza sativa* L.). Biotechnol. Lett..

[69] Joo J., Choi H.J., Lee Y.H., Kim Y.K., Song S.I. (2013). A transcriptional repressor of the ERF family confers drought tolerance to rice and regulates genes preferentially located on chromosome 11. Planta.

[70] Zhang H., Zhang J., Quan R., Pan X., Wan L., Huang R. (2013). EAR motif mutation of rice OsERF3 alters the regulation of ethylene biosynthesis and drought tolerance. Planta.

[71] Jung H., Chung P.J., Park S.-H., Redillas M.C.F.R., Kim Y.S., Suh J.-W., Kim J.-K. (2017). Overexpression of *OsERF48* causes regulation of *OsCML16*, a calmodulin-like protein gene that enhances root growth and drought tolerance. Plant Biotechnol. J..

[72] Xiong H., Yu J., Miao J., Li J., Zhang H., Wang X., Liu P., Zhao Y., Jiang C., Yin Z. (2018). Natural variation in *OsLG3* increases drought tolerance in rice by inducing ROS scavenging. Plant Physiol..

[73] Jin Y., Pan W., Zheng X., Cheng X., Liu M., Ma H., Ge X. (2018). OsERF101, an ERF family transcription factor, regulates drought stress response in reproductive tissues. Plant Mol. Biol..

[74] Lee D.-K., Jung H., Jang G., Jeong J.S., Kim Y.S., Ha S.-H., Choi Y.D., Kim J.-K. (2016). Overexpression of the *OsERF71* transcription factor alters rice root structure and drought resistance. Plant Physiol..

[75] Li J., Guo X., Zhang M., Wang X., Zhao Y., Yin Z., Zhang Z., Wang Y., Xiong H., Zhang H. (2018). OsERF71 confers drought tolerance via modulating ABA signaling and proline biosynthesis. Plant Sci..

[76] Ahn H., Jung I., Shin S.J., Park J., Rhee S., Kim J.K., Jung W., Kwon H.-B., Kim S. (2017). Transcriptional network analysis reveals drought resistance mechanisms of AP2/ERF transgenic rice. Front. Plant Sci..

[77] Jung S.E., Bang S.W., Kim S.H., Seo J.S., Yoon H.B., Kim Y.S., Kim J. (2021). Overexpression of *OsERF83*, a vascular tissue-specific tran-scription factor gene, confers drought tolerance in rice. Int. J. Mol. Sci..

[78] Park S.I., Kwon H.J., Cho M.H., Song J.S., Kim B.G., Baek J., Kim S.L., Ji H.S., Kwon T., Kim K. (2021). The *OsERF115*/*AP2EREBP110* transcription factor is involved in the multiple stress tolerance to heat and drought in rice plants. Int. J. Mol. Sci..

[79] Yaish M.W., El-Kereamy A., Zhu T., Beatty P.H., Good A.G., Bi Y.-M., Rothstein S.J. (2010). The APETALA-2-like transcription factor OsAP2-39 controls key interactions between abscisic acid and gibberellin in rice. PLoS Genet..

[80] Wan L., Zhang J., Zhang H., Zhang Z., Quan R., Zhou S.-R., Huang R. (2011). Transcriptional activation of *OsDERF1* in OsERF3 and OsAP2-39 negatively modulates ethylene synthesis and drought tolerance in rice. PLoS ONE.

[81] Yu Y., Yang D., Zhou S., Gu J., Wang F., Dong J., Huang R. (2017). The ethylene response factor OsERF109 negatively affects ethylene biosynthesis and drought tolerance in rice. Protoplasma.

[82] Zhang Y., Li J., Chen S., Ma X., Wei H., Chen C., Gao N., Zou Y., Kong D., Li T. (2020). An APETALA2/ethylene responsive factor, *OsEBP89* knockout enhances adaptation to direct-seeding on wet land and tolerance to drought stress in rice. Mol. Genet. Genom..

[83] Huang L., Wang Y., Wang W., Zhao X., Qin Q., Sun F., Hu F., Zhao Y., Li Z., Fu B. (2018). Characterization of transcription factor gene *OsDRAP1* conferring drought tolerance in rice. Front. Plant Sci..

[84] Wang Y., Wang J., Zhao X., Yang S., Huang L., Du F., Li Z., Zhao X., Fu B., Wang W. (2020). Overexpression of the transcription factor gene *OsSTAP1* increases salt tolerance in iice. Rice.

[85] Wang Y., Huang L., Du F., Wang J., Zhao X., Li Z., Wang W., Xu J., Fu B. (2021). Comparative transcriptome and metabolome profiling reveal molecular mechanisms underlying OsDRAP1-mediated salt tolerance in rice. Sci. Rep..

[86] Huang S., Ma Z., Hu L., Huang K., Zhang M., Zhang S., Jiang W., Wu T., Du X. (2021). Involvement of rice transcription factor OsERF19 in response to ABA and salt stress responses. Plant Physiol. Biochem..

[87] Chen H.C., Chien T.C., Chen T.Y., Chiang M.H., Lai M.H., Chang M.C. (2021). Overexpression of a novel ERF-X-type transcription factor, *OsERF106MZ*, reduces shoot growth and tolerance to salinity stress in rice. Rice.

[88] Serra T.S., Figueiredo D.D., Cordeiro A.M., Almeida D.M., Lourenço T., Abreu I.A., Sebastián A., Fernandes L., Contreras-Moreira B., Oliveira M.M. (2013). *OsRMC*, a negative regulator of salt stress response in rice, is regulated by two AP2/ERF transcription factors. Plant Mol. Biol..

[89] Ito Y., Katsura K., Maruyama K., Taji T., Kobayashi M., Seki M., Shinozaki K., Yamaguchi-Shinozaki K. (2006). Functional analysis of rice DREB1/CBF-type transcription factors involved in cold-responsive gene expression in transgenic rice. Plant Cell Physiol..

[90] Fu X.Y., Zhang Z., Peng R.H., Xiong A.S., Liu J.G., Wu L.J., Gao F., Zhu H., Guo Z., Yao Q. (2007). Isolation and characterization of a novel cDNA encoding ERF/AP2-type transcription factor OsAP25 from *Oryza sativa* L.. Biotechnol. Lett..

[91] Kong W., Zhang C., Qiang Y., Zhong H., Zhao G., Li Y. (2020). Integrated RNA-seq analysis and meta-QTLs mapping provide insights into cold stress response in rice seedling roots. Int. J. Mol. Sci..

[92] Moon S.-J., Min M.K., Kim J.-A., Kim D.Y., Yoon I.S., Kwon T.R., Byun M.O., Kim B.-G. (2019). Ectopic expression of *OsDREB1G*, a member of the OsDREB1 subfamily, confers cold stress tolerance in rice. Front. Plant Sci..

[93] Ogawa T., Yamamoto T., Khush G.S., Mew T.W., Kaku H. (1988). Near isogenic lines as international differentials for resistance to bacterial blight of rice. Rice Genet. Newsl..

[94] Young S.A., Guo A., Guikema J.A., White F.F., Leach J.E. (1995). Rice cationic peroxidase accumulates in xylem vessels during incompatible interactions with *Xanthomonas oryzae* pv. *oryzae*. Plant Physiol..

[95] Wang H., Hao J., Chen X., Hao Z., Wang X., Lou Y., Peng Y., Guo Z. (2007). Overexpression of rice *WRKY89* enhances ultraviolet B tolerance and disease resistance in rice plants. Plant Mol. Biol..

[96] Kishi-Kaboshi M., Okada K., Kurimoto L., Murakami S., Umezawa T., Shibuya N., Yamane H., Miyao A., Takatsuji H., Takahashi A. (2010). A rice fungal MAMP-responsive MAPK cascade regulates metabolic flow to antimicrobial metabolite synthesis. Plant J..

[97] Shen X., Yuan B., Liu H., Li X., Xu C., Wang S. (2010). Opposite functions of a rice mitogen-activated protein kinase during the process of resistance against *Xanthomonas oryzae*. Plant J..

[98] Wang Q., Li J., Hu L., Zhang T., Zhang G., Lou Y. (2013). OsMPK3 positively regulates the JA signaling pathway and plant resistance to a chewing herbivore in rice. Plant Cell Rep..

[99] Jalmi S.K., Sinha A.K. (2016). Functional involvement of a mitogen activated protein kinase module, OsMKK3-OsMPK7-OsWRK30 in mediating resistance against *Xanthomonas oryzae* in rice. Sci. Rep..

[100] Zhou P., Chen M., Zhang Y., Gao Q., Noman A., Wang Q., Li H., Lu J., Lou Y. (2019). OsMKK3, a stress-responsive protein kinase, positively regulates rice resistance to *Nilaparvata lugens* via phytohormone dynamics. Int. J. Mol. Sci..

[101] Nuruzzaman M., Gupta M., Zhang C., Wang L., Xie W., Xiong L., Zhang Q., Lian X. (2008). Sequence and expression analysis of the thioredoxin protein gene family in rice. Mol. Genet. Genom..

[102] Kim S.H., Kim J.Y., Kim S.J., An K.S., An G., Kim S.R. (2007). Isolation of cold stress-responsive genes in the reproductive organs, and characterization of the *OsLti6b* gene from rice (*Oryza sativa* L.). Plant Cell Rep..

[103] Morsy M.R., Almutairi A.M., Gibbons J., Yun S.J., de Los Reyes B.G. (2005). The *OsLti6* genes encoding low-molecular weight membrane proteins are differentially expressed in rice cultivars with contrasting sensitivity to low temperature. Gene.

[104] Saijo Y., Hata S., Kyozuka J., Shimamoto K., Izui K. (2000). Overexpression of a single Ca^2+^-dependent protein kinase confers both cold and salt/drought tolerance on rice plants. Plant J..

[105] Su C.F., Wang Y.C., Hsieh T.H., Lu C.A., Tseng T.H., Yu S.M. (2010). A novel MYBS3-dependent pathway confers cold tolerance in rice. Plant Physiol..

[106] Vishwakarma K., Upadhyay N., Kumar N., Yadav G., Singh J., Mishra R.K., Kumar V., Verma R., Upadhyay R.G., Pandey M. (2017). Abscisic acid signaling and abiotic stress tolerance in plants: A review on current knowledge and future prospects. Front. Plant Sci..

[107] Kazan K. (2015). Diverse roles of jasmonates and ethylene in abiotic stress tolerance. Trends Plant Sci..

[108] Zhao H., Yin C.C., Ma B., Chen S.Y., Zhang J.S. (2021). Ethylene signaling in rice and Arabidopsis: New regulators and mechanisms. J. Integr. Plant Biol..

[109] Beyer E.M. (1976). A potent inhibitor of ethylene action in plants. Plant Physiol..

[110] Zhang H., Huang L., Dai Y., Liu S., Hong Y., Tian L., Huang L., Cao Z., Li D., Song F. (2015). Arabidopsis AtERF15 positively regulates immunity against *Pseudomonas syringae* pv. *tomato* DC3000 and *Botrytis cinerea*. Front. Plant Sci..

[111] Catinot J., Huang J.B., Huang P.Y., Tseng M.Y., Chen Y.L., Gu S.Y., Lo W., Wang L., Chen Y., Zimmerli L. (2015). ETHYLENE RE-SPONSE FACTOR 96 positively regulates Arabidopsis resistance to necrotrophic pathogens by direct binding to GCC elements of jasmonate- and ethylene-responsive defence genes. Plant Cell Environ..

[112] Yamamoto T., Yoshida Y., Nakajima K., Tominaga M., Gyohda A., Suzuki H., Okamoto T., Nishimura T., Yokotani N., Minami E. (2018). Expression of *RSOsPR10* in rice roots is antagonistically regulated by jasmonate/ethylene and salicylic acid via the activator OsERF87 and the repressor OsWRKY76, respectively. Plant Direct.

[113] Yang D.L., Yang Y., He Z. (2013). Roles of plant hormones and their interplay in rice immunity. Mol. Plant.

[114] Liu W., Liu J., Triplett L., Leach J.E., Wang G.L. (2014). Novel insights into rice innate immunity against bacterial and fungal pathogens. Annu. Rev. Phytopathol..

[115] Nasir F., Tian L., Chang C., Li X., Gao Y., Tran L.S.P., Tian C. (2018). Current understanding of pattern-triggered immunity and hormone-mediated defense in rice (*Oryza sativa*) in response to *Magnaporthe oryzae* infection. Semin. Cell Dev. Biol..

[116] Underwood W. (2012). The plant cell wall: A dynamic barrier against pathogen invasion. Front. Plant Sci..

[117] Bacete L., Mélida H., Miedes E., Molina A. (2018). Plant cell wall-mediated immunity: Cell wall changes trigger disease resistance responses. Plant J..

[118] Malinovsky F.G., Fangel J.U., Willats W.G. (2014). The role of the cell wall in plant immunity. Front. Plant Sci..

[119] Li W., Zhong S., Li G., Li Q., Mao B., Deng Y., Zhang H., Zeng L., Song F., He Z. (2011). Rice RING protein OsBBI1 with E3 ligase activity confers broad-spectrum resistance against *Magnaporthe oryzae* by modifying the cell wall defence. Cell Res..

[120] Hu K., Cao J., Zhang J., Xia F., Ke Y., Zhang H., Xie W., Liu H., Cui Y., Cao Y. (2017). Improvement of multiple agronomic traits by a disease resistance gene via cell wall reinforcement. Nat. Plants.

[121] Cao Y., Zhang Y., Chen Y., Yu N., Liaqat S., Wu W., Chen D., Cheng S., Wei X., Cao L. (2021). *OsPG1* encodes a polygalacturonase that determines cell wall architecture and affects resistance to bacterial blight pathogen in rice. Rice.

[122] Wang D., Yuan S., Yin L., Zhao J., Guo B., Lan J., Li X. (2012). A missense mutation in the transmembrane domain of *CESA9* affects cell wall biosynthesis and plant growth in rice. Plant Sci..

[123] Song X.-Q., Liu L.-F., Jiang Y.-J., Zhang B.-C., Gao Y.-P., Liu X.-L., Lin Q.-S., Ling H.-Q., Zhou Y.-H. (2013). Disruption of secondary wall cellulose biosynthesis alters cadmium translocation and tolerance in rice plants. Mol. Plant.

[124] Huang Y., Jiao Y., Xie N., Guo Y., Zhang F., Xiang Z., Wang R., Wang F., Gao Q., Tian L. (2019). *OsNCED5*, a 9-*cis*-epoxycarotenoid dioxygenase gene, regulates salt and water stress tolerance and leaf senescence in rice. Plant Sci..

[125] Huang Y., Guo Y., Liu Y., Zhang F., Wang Z., Wang H., Wang F., Li D., Mao D., Luan S. (2018). 9-*cis*-epoxycarotenoid dioxygenase 3 regulates plant growth and enhances multi-abiotic stress tolerance in rice. Front. Plant Sci..

[126] Sun X.L., Cao Y.L., Yang Z.F., Xu C.G., Li X.H., Wang S.P., Zhang Q. (2004). *Xa26*, a gene conferring resistance to *Xanthomonas oryzae* pv. *oryzae* in rice, encodes an LRR receptor kinase-like protein. Plant J..

[127] Hong Y., Zhang H., Huang L., Li D., Song F. (2016). Overexpression of a stress-responsive NAC transcription factor gene *ONAC022* improves drought and salt tolerance in rice. Front. Plant Sci..

[128] Huang L., Hong Y., Zhang H., Li D., Song F. (2016). Rice NAC transcription factor ONAC095 plays opposite roles in drought and cold stress tolerance. BMC Plant Biol..

[129] Zhang J., Peng Y.L., Guo Z.J. (2008). Consititutive expression of pathogen-inducible *OsWRKY31* enhance disease resistance and affects root growth and auxin response in transgenic rice plants. Cell Res..

[130] Hong Y., Yang Y., Zhang H., Huang L., Li D., Song F. (2017). Overexpression of *MoSM1*, encoding for an immunity-inducing protein from *Magnaporthe oryzae*, in rice confers broad-spectrum resistance against fungal and bacterial diseases. Sci. Rep..

[131] Peng Y.L., Shishiyama J. (1988). Temporal sequence of cytological events in rice leaves affected with *Pyricularia oryzae*. Can. J. Bot..

[132] Qi M., Yang Y.N. (2002). Quantification of *Magnaporthe grisea* during infection of rice plants using real-time polymerase chain re-action and northern blot/phosphoimaging analyses. Phytopathology.

[133] Qiu D.Y., Xiao J., Ding X.H., Xiong M., Cai M., Cao Y.L., Li X., Xu C., Wang S. (2007). OsWRKY13 mediates rice disease resistance by regulating defense-related genes in salicylate-and jasmonate-dependent signaling. Mol. Plant-Microbe Interact..

[134] Bates L.S., Waldren R.P., Teare I.D. (1973). Rapid determination of free proline for water-stress studies. Plant Soil.

[135] Kim S.G., Kim K.W., Park E.W., Choi D. (2002). Silicon-induced cell wall fortification of rice leaves: A possible cellular mechanism of enhanced host resistance to blast. Phytopathology.

