# Supplementary material for: ERF Transcription Factor OsBIERF3 Positively Contributes to Immunity against Fungal and Bacterial Diseases but Negatively Regulates Cold Tolerance in Rice
Source: Int J Mol Sci. 2022 Jan 6;23(2):606. doi: 10.3390/ijms23020606 (PMC8775505; doi:10.3390/ijms23020606)
Supplement: Supplementary file 1 [file ijms-23-00606-s001.zip › ijms-1497401-supplementary.pdf]

Supplementary Materials

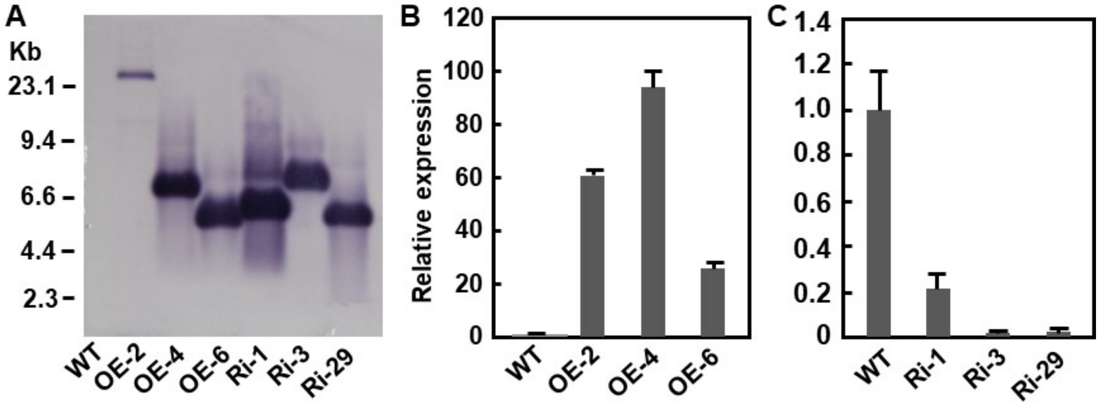

Figure S1. Molecular characterization of the OsBIERF3-OE and OsBIERF3-Ri lines. (**A**) Southern blot analysis of the copy number in independent transgenic lines. DNA from T3 generation transgenic and nontransgenic WT plants was digested with *Eco*RI, separated on an agarose gel, transferred to a Hybond-N^+ nylon membrane and hybridized with a DIG-labelled fragment amplified from the *HptII* gene as a probe. (**B**) Expression levels of *OsBIERF3* in OsBIERF3-OE and nontransgenic WT plants. (**C**) Expression levels of *OsBIERF3* in OsBIERF3-RNAi and nontransgenic WT plants. Leaf samples were collected from 8-week-old plants grown in greenhouse and expression of *OsBIERF3* was analyzed by qRT-PCR. A rice *Actin* gene was used as an internal control and the expression level of *OsBIERF3* in WT plants was set as 1.0. Data presented in (**B**) and (**C**) are the means ± SD from three independent experiments and different letters on columns indicate statistically significant difference at $p < 0.05$ level.

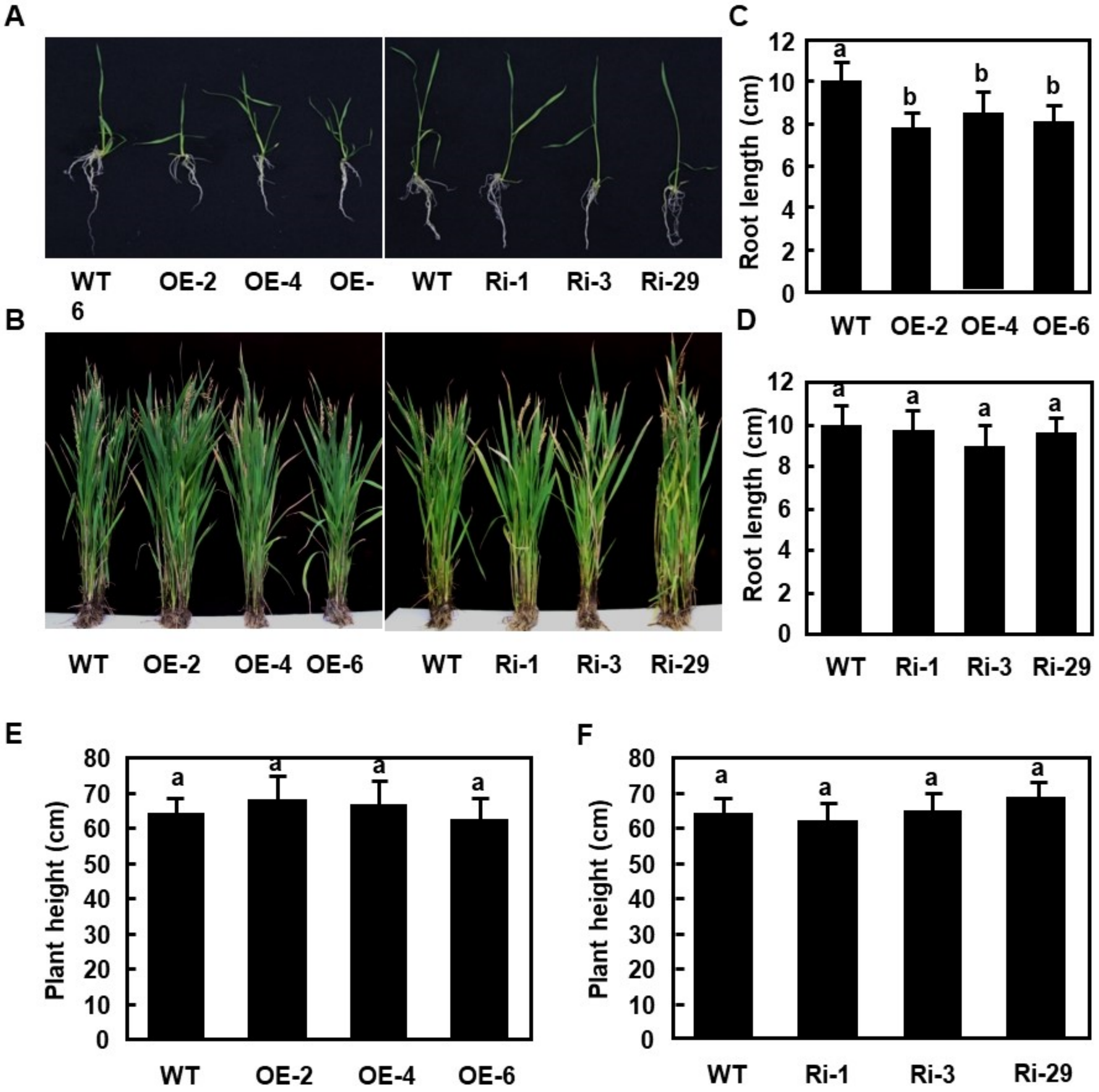

Figure S2. Growth phenotype of the OsBIERF3-OE and OsBIERF3-RNAi plants at seedling and adult stages. (**A**) Growth phenotype of 3-week-old seedlings. (**B**) Growth phenotype of adult plants at heading stage. (**C**) and (**D**) Root length of the OsBIERF3-OE and OsBIEF3-RNAi seedlings. (**E**) and (**F**) Height of the OsBIERF3-OE and OsBIEF3-RNAi adult plants. Experiments were repeated three times with similar results and data from one representative experiment were shown in (**A**) and (**B**). Data presented in (**C–F**) are the means ± SD from three independent experiments and different letters on columns indicate statistically significant difference at $p < 0.05$ level.

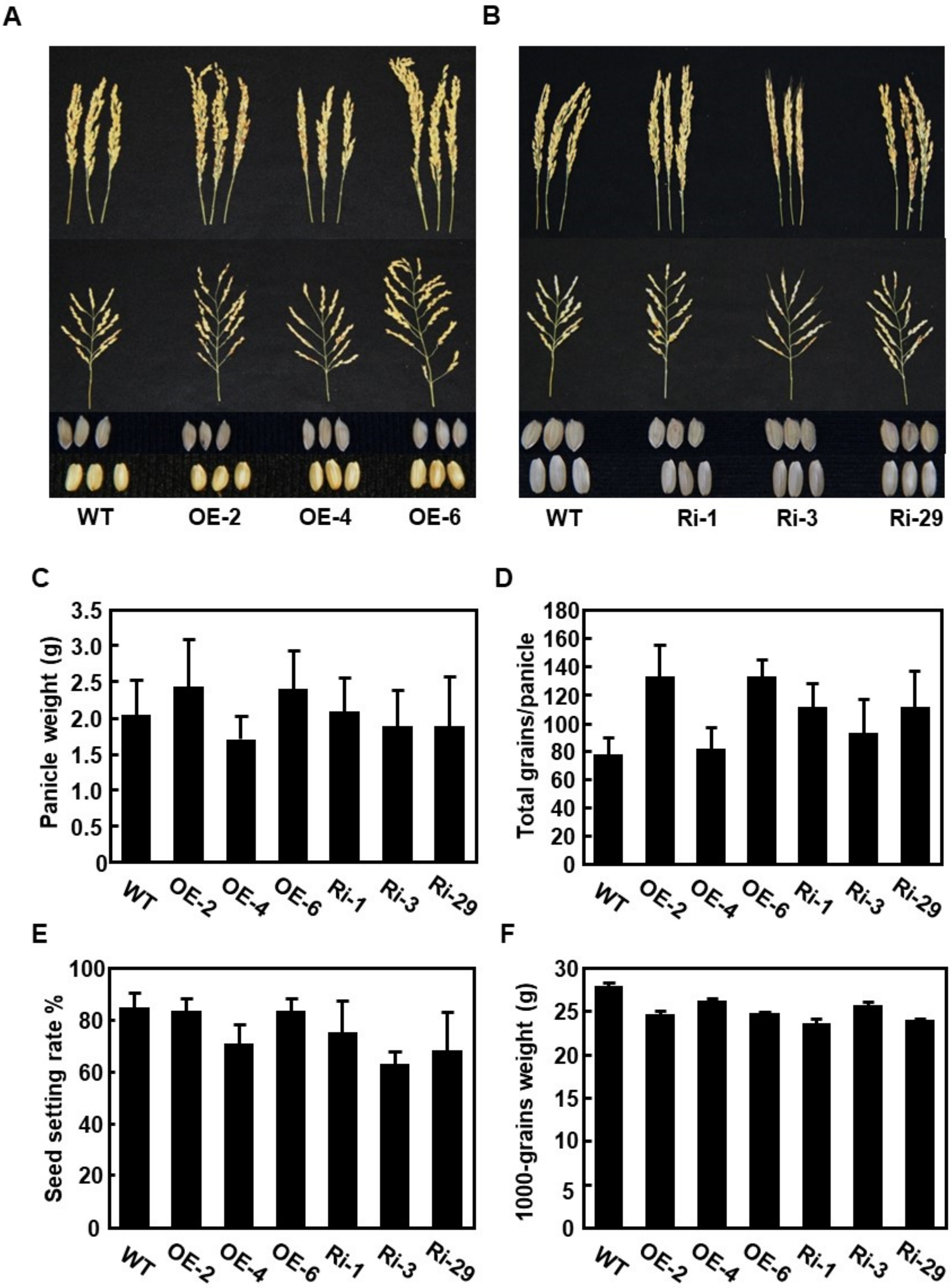

Figure S3. Panicle and grain phenotypes of the OsBIERF3-OE and OsBIERF3-RNAi plants. (**A**) and (**B**) Comparison of the panicles and grain sizes from the OsBIERF3-OE and OsBIERF3-RNAi plants grown in a greenhouse. (**C**) Panicle weight; (**D**) Total grains per panicle; (**E**) Seed setting rate; (**F**) 1000-grains weight of the OsBIERF3-OE and OsBIERF3-RNAi plants grown in a greenhouse. Experiments were repeated three times with similar results and data from one representative experiment were shown in (**A**) and (**B**). Data presented in (**C–F**) are the means ± SD from three independent experiments and different letters on columns indicate statistically significant difference at $p < 0.05$ level.

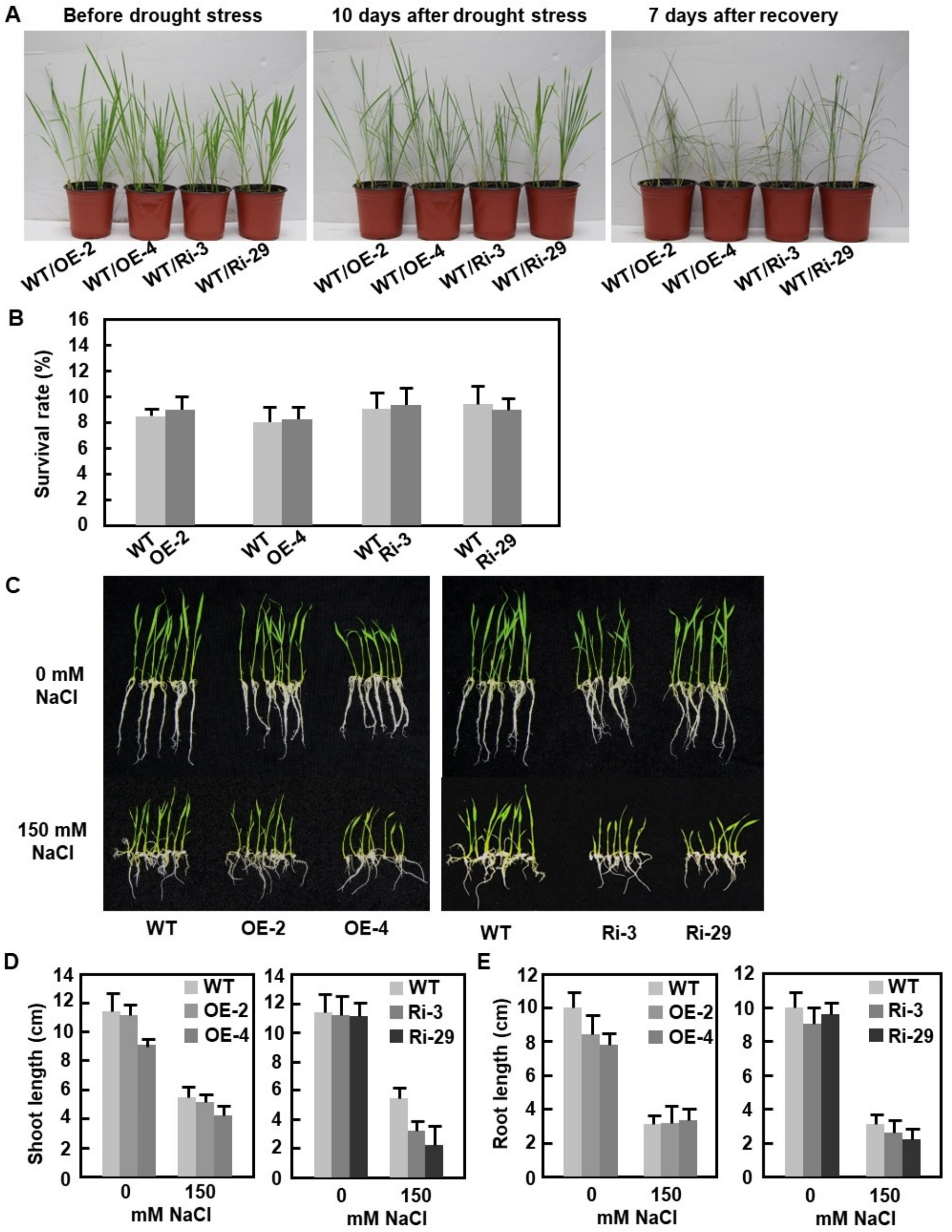

Figure S4. OsBIERF3 is not involved in rice drought and salt tolerance. (**A**) Growth performance and phenotype of the WT, OsBIERF3-OE and OsBIERF3-Ri plants before stress, 10 days after water withholding, and 7 days after re-watering. (**B**) Survival rate of the WT, OsBIERF3-OE and OsBIERF3-Ri plants at 7 days after recovery. (**C**) Phenotype of two-week-old WT, OsBIERF3-OE and OsBIERF3-Ri seedlings grown on 1/2 MS medium without or with 150 mM NaCl. (**D**) Shoot length of the WT, OsBIERF3-OE and OsBIERF3-Ri seedlings as in (**C**). (**E**) Root length of the WT, OsBIERF3-OE and OsBIERF3-Ri seedlings as in (**C**). Experiments were repeated three times with similar results and data from one representative experiment were shown in (**A**) and (**B**). Data presented in (**B**,**D**,**E**) are the means ± SD from three independent experiments.

Table S1. List of upregulated genes in OsBIERF3-OE plants.

Locus	Description	Fold
Defense genes		
LOC_Os02g41904	DEF7-Defensin and Defensin-like DEFL family	189.742
LOC_Os06g47600	Thaumatin family domain, PR5	15.732
LOC_Os01g62260	Thaumatin, PR5	11.232
LOC_Os12g38120	Thaumatin family domain, PR5	5.490
LOC_Os10g05660	Thaumatin, putative, PR5	5.197
LOC_Os04g39150	Pathogenesis-related Bet v I family protein, PR10	2.572
LOC_Os12g38150	Osmotin, putative, PR5	2.568
LOC_Os11g41170	Disease resistance protein RPM1	2.295
LOC_Os10g27280	Thaumatin, putative, PR5	2.252
LOC_Os11g12340	Disease resistance protein RPM1	5.202
LOC_Os12g36810	Polygalacturonase	2.195
LRR proteins		
LOC_Os07g31840	Leucine-rich repeat family protein	2.410
LOC_Os04g48760	Leucine-rich repeat family protein	9.560
LOC_Os12g44090	Leucine-rich repeat family protein	6.587
LOC_Os04g08390	Leucine Rich Repeat family protein	2.008
LOC_Os03g03570	Leucine-rich repeat transmembrane protein kinase	2.895
LOC_Os07g05190	Leucine-rich repeat family protein	12.943
LOC_Os02g58030	Leucine-rich repeat-containing protein 40	2.160
LOC_Os11g42060	Leucine Rich Repeat family protein	2.062
LOC_Os12g40860	Leucine Rich Repeat family protein	2.641
LOC_Os07g18560	LRR and FBD domain containing protein	3.363
LOC_Os08g09750	F-box domain and LRR containing protein	2.563
LOC_Os11g39310	NB-ARC domain containing protein	32.444
LOC_Os12g10710	NB-ARC domain containing protein	9.357
LOC_Os11g39190	NB-ARC domain containing protein	101.595
LOC_Os01g04720	leucine-rich repeat protein-related	2.020
LOC_Os11g46210	NB-ARC domain containing protein	29.802
Protein kinases		
LOC_Os04g58700	Kinase activity, expressed protein	2.106
LOC_Os06g10230	Receptor-like protein kinase 5 precursor	3.754
LOC_Os03g64050	Receptor protein kinase	2.086
LOC_Os01g70970	Protein kinase	3.603
LOC_Os08g34650	Receptor-like protein kinase precursor	2.255
LOC_Os01g53840	Protein kinase family protein	2.992
LOC_Os12g41180	LSTK-1-like kinase	3.175
LOC_Os01g12430	Receptor-like protein kinase	2.142
LOC_Os01g60670	Receptor-like protein kinase precursor	6.827
LOC_Os03g61010	Protein kinase family protein	2.186
LOC_Os01g44110	Serine/threonine-protein kinase	2.061
LOC_Os01g12390	Inactive receptor kinase At2g26730 precursor	2.319
LOC_Os05g01040	Serine/threonine-protein kinase	2.217

LOC_Os01g07940	AGC_PVPK_like_kin82y.3	2.019
LOC_Os06g03970	Receptor-like protein kinase 5 precursor	6.267
LOC_Os01g04230	Inactive receptor kinase At2g26730 precursor	4.561
LOC_Os10g41390	Protein kinase domain containing protein	2.742
LOC_Os05g39080	Protein kinase family protein	3.327
LOC_Os03g04490	Cyclin-dependent kinase inhibitor	2.017
LOC_Os04g54200	Diacylglycerol kinase	3.355
LOC_Os06g50910	Phosphatidylinositol kinase and FAT protein	2.017
LOC_Os05g11750	Protein kinase	7.843
LOC_Os08g34380	Receptor-like kinase	2.171
LOC_Os03g21730	Receptor-like protein kinase precursor	3.469
LOC_Os05g39080	Protein kinase family protein	3.612
LOC_Os04g59320	Protein kinase domain containing protein	2.958
LOC_Os04g20810	Receptor protein kinase	2.737
LOC_Os02g58390	Inactive receptor kinase At2g26730 precursor	2.470
LOC_Os08g17410	BRASSINOSTEROID INSENSITIVE 1 precursor	2.211
LOC_Os03g57780	Protein kinase	6.145
LOC_Os03g18630	Receptor-like kinase RHG1	3.481
LOC_Os07g06570	Receptor protein kinase CRINKLY4 precursor	3.751
LOC_Os08g39590	Inactive receptor kinase At2g26730 precursor	7.279
LOC_Os11g47030	Receptor protein kinase///receptor-like protein kinase precursor	2.728
LOC_Os05g30820	Protein kinase	6.555
LOC_Os05g40050	Receptor-like protein kinase 2 precursor	3.588
LOC_Os03g51440	LRR receptor-like protein kinase	3.107
LOC_Os03g50810	Receptor protein kinase TMK1 precursor	2.311
LOC_Os01g60330	Inactive receptor kinase At2g26730 precursor	3.101
LOC_Os08g39240	OsWAK76 receptor-like cytoplasmic kinase	2.847
LOC_Os04g56430	Cysteine-rich receptor-like protein kinas	2.147
LOC_Os12g13380	Adenylate kinase	2.241
LOC_Os08g02120	PfkB-like carbohydrate kinase family protein	3.379
LOC_Os07g44630	Thymidylate kinase	4.481
LOC_Os03g46910	Pyruvate kinase	2.688
LOC_Os12g05120	Receptor kinase	4.209
MAPK genes		
LOC_Os03g43590	MAPKK-RELATED SERINE/THREONINE PROTEIN KINASES, LSTK-1-like kinase	3.339
LOC_Os03g43590	MAPKK-RELATED SERINE/THREONINE PROTEIN KINASES, LSTK-1-like kinase	3.339
LOC_Os09g21510	MAPKKK3, STE_MEKK_ste11_MAP3K.2 - STE kinases include homologs to sterile 7	11.588
LOC_Os02g35010	STE_MEKK_ste11_MAP3K.9 - STE kinases include homologs to sterile 7	2.994
LOC_Os08g32600	STE_MEKK_ste11_MAP3K.21 - STE kinases include homologs to sterile 7	13.472

LOC_Os06g27890	(OsMEK3) STE_MEK_ste7_MAP2K.8-STE kinases include homologs to sterile 7	6.278
LOC_Os06g06090	(MAPK6)CGMC_MAPKCMGC_2_ERK.12-CGMC includes CDA, MAPK, GSK3, and CLKC kinases	5.867
LOC_Os03g17700	(OsMPK3)CGMC_MAPKCGMC_2_ERK.2-CGMC includes CDA, MAPK, GSK3, and CLKC kinases	4.122
Zinc fingers		
LOC_Os07g40480	Zinc finger family protein	3.413
LOC_Os02g10920	Zinc finger family protein	2.787
LOC_Os03g07790	Zinc finger, C3HC4 type domain containing protein	2.571
LOC_Os03g03550	bZIP family transcription factor	2.299
LOC_Os10g19180	Zinc finger family protein	7.213
LOC_Os01g70100	Zinc finger DHHC domain-containing protein	3.229
LOC_Os08g44050	ZOS8-12 - C2H2 zinc finger protein	18.240
LOC_Os05g36090	Zinc finger DHHC domain-containing protein	2.270
LOC_Os10g39850	Zinc finger, C3HC4 protein	6.113
LOC_Os03g21800	bZIP transcription factor family protein	2.316
LOC_Os01g54210	GATA zinc finger domain containing protein	3.594
LOC_Os01g11350	bZIP transcription factor domain containing protein	2.236
LOC_Os01g57650	ZOS1-12 - C2H2 zinc finger protein	4.904
LOC_Os02g10920	Zinc finger family protein	3.998
LOC_Os02g47810	Dof zinc finger domain containing protein	2.165
LOC_Os10g40810	GATA zinc finger domain containing protein	3.602
LOC_Os05g32350	Zinc finger family protein	3.185
LOC_Os12g16690	Zinc finger, C3HC4 type domain containing protein	3.905
LOC_Os05g01230	Zinc finger, C3HC4 type domain containing protein	3.519
LOC_Os06g46910	ZOS6-07 - C2H2 zinc finger protein	5.168
LOC_Os02g44090	Zinc finger protein	2.272
LOC_Os09g12720	Zinc finger, C3HC4 type domain containing protein	2.171
LOC_Os12g16210	Zinc finger family protein	17.620
LOC_Os03g21800	bZIP transcription factor family protein	2.130
LOC_Os02g14910	bZIP transcription factor family protein	2.614
LOC_Os12g09250	bZIP transcription factor domain containing protein	2.172
LOC_Os12g42250	ZOS12-10 - C2H2 zinc finger protein	2.282
LOC_Os05g44400	GATA zinc finger domain containing protein	6.130
LOC_Os05g51830	ZOS5-12 - C2H2 zinc finger protein	2.733
LOC_Os10g40810	GATA zinc finger domain containing protein, transcription factor	6.116
LOC_Os05g01610	FYVE zinc finger domain containing protein	2.081
ERFs		
LOC_Os01g12440	AP2 domain containing protein	2.130
LOC_Os05g32270	ERF, AP2 domain transcription factor protein	3.957
LOC_Os01g01600	Ethylene-responsive transcription factor	2.180

LOC_Os06g01780	Ethylene-responsive element-binding protein	2.105
LOC_Os04g55970	AP2-like ethylene-responsive transcription factor AINTEGUMENTA	2.136
MYBs		
LOC_Os01g12860	MYB family transcription factor	6.532
LOC_Os08g33660	MYB family transcription factor	13.769
LOC_Os12g13570	MYB family transcription factor	3.504
NACs		
LOC_Os08g02300	NAC, no apical meristem transcription factor protein	2.351
LOC_Os05g34310	NAC, no apical meristem transcription factor protein	2.513
LOC_Os02g38130	NAC, no apical meristem transcription factor protein	2.167
WRKYs		
LOC_Os02g43560	WRKY34	4.248
LOC_Os01g74140	WRKY17	3.237
Other TFs		
LOC_Os02g50630	E2F family transcription factor protein	3.782
LOC_Os03g57190	TCP family transcription factor	2.549
LOC_Os01g11550	TCP family transcription factor	13.059
LOC_Os05g49780	Histone-like transcription factor and archaeal histone	2.433
LOC_Os01g55100	TCP3, sequence-specific transcription factor	2.357
LOC_Os03g58830	BEE 1 sequence-specific transcription factor	6.911
LOC_Os09g33580	BEE 1, sequence-specific transcription factor	2.107
LOC_Os07g39800	Transcription repressor HOTR	2.314
LOC_Os06g13670	E2F family transcription factor protein	5.198
LOC_Os01g08190	Transcriptional corepressor LEUNIG	2.613
LOC_Os06g50900	Sequence-specific DNA binding transcription factor	3.420
LOC_Os02g01940	Transcription factor jumonji	3.003
LOC_Os03g63400	Transcription factor BTF3	3.122
LOC_Os08g42470	BEE 1, DNA binding transcription factor	7.663
LOC_Os12g07950	Transcriptional regulator Sir2 family protein	8.632
Helix-loop-helix		
LOC_Os05g27090	Basic helix-loop-helix domain containing protein	2.570
LOC_Os05g27090	Basic helix-loop-helix domain containing protein	2.570
LOC_Os03g51910	Basic helix-loop-helix protein	2.937
LOC_Os03g18210	Basic helix-loop-helix	2.090
LOC_Os02g51320	Helix-loop-helix DNA-binding domain containing protein, response to abiotic stimulus	3.539
LOC_Os06g41060	Helix-loop-helix DNA-binding	2.631
LOC_Os10g26410	bHLH family protein	2.281
LOC_Os02g49480	Helix-loop-helix DNA-binding domain protein	4.480
LOC_Os07g47960	Basic helix-loop-helix domain protein	2.067
LOC_Os05g46370	bHelix-loop-helix transcription factor	2.676
P450s		
LOC_Os03g04530	Cytochrome P450	51.042

LOC_Os10g08474	Cytochrome P450	27.056
LOC_Os03g25490	Cytochrome P450 72A1	2.277
LOC_Os01g72740	Cytochrome P450	2.153
LOC_Os07g11739	Cytochrome P450	2.061
LOC_Os01g72760	Cytochrome P450	3.629
LOC_Os02g44654	Cytochrome P450	2.740
Cell wall synthases		
LOC_Os03g56060	CSLC9 - cellulose synthase-like family C	2.981
LOC_Os06g42020	CSLA9 - cellulose synthase-like family A	8.0402
LOC_Os07g43710	CSLA7 - cellulose synthase-like family A	2.0394
LOC_Os09g09980	Glucan endo-1,3-beta-glucosidase	2.2617
LOC_Os10g20650	Glucan endo-1,3-beta-glucosidase-related	6.5595
LOC_Os03g12140	Glucan endo-1,3-beta-glucosidase precursor	11.2971
LOC_Os07g35480	Glucan endo-1,3-beta-glucosidase precursor	3.24
LOC_Os07g35520	Glucan endo-1,3-beta-glucosidase precursor	3.5807
LOC_Os11g36940	Glucan endo-1,3-beta-glucosidase precursor	2.5349
LOC_Os05g43690	Glucan endo-1,3-beta-glucosidase-like protein 3	21.6953
Ubiquitin enzymes		
LOC_Os09g39500	Ubiquitin fusion protein	2.4319
LOC_Os01g42040	Ubiquitin-conjugating enzyme, E2	4.8042
LOC_Os01g16650	Ubiquitin-conjugating enzyme, E2	28.2254
LOC_Os04g49130	Ubiquitin-conjugating enzyme, E2	3.8143
LOC_Os02g38410	Ubiquitin family protein	2.7093
Others		
LOC_Os04g59260	Peroxidase precursor	10.7702
LOC_Os10g28060	3-ketoacyl-CoA synthase	22.4257
LOC_Os11g05290	Stress responsive A/B Barrel domain protein	14.8384
LOC_Os01g05900	Core histone H2A/H2B/H3/H4 domain containing	2.1016

Table S2. Primers used in this study.

Name	Primer Sequence
Plasmid construction	
OsBIERF3-F-OE	ATA GGATCC ATGCTGCTTAATCCGGCGTC
OsBIERF3-R-OE	ATA CCTAGG TTAGCTCACCAGCTGCTGGA
OsBIERF3-F-Ri	ATA CCGC GGATCC ACAGCATCCGGCACCACCTC
OsBIERF3-R-Ri	GCG CCATGG CTGCAG ACGGCCTCGCCCTCGCCCTC
OsBIERF3-GFP-F	CGGGATCCATGCTGCTTAATCCGGCGTC
OsBIERF3-GFP-R	GCTCTAGATTAGCTCACCAGCTGCTGGA
OsBIERF3-pBD-F	CGGAATTC ATGCTGCTTAATCCGGCGTC
OsBIERF3-pBD-R	AACTGCAG TTAGCTCACCAGCTGCTGGA
OsBIERF3ΔN-BKT7-F	CGGAATTCGTGGCGTCGAGGGGGAAG
OsBIERF3ΔC-BKT7-R	CGGGATCCCGCCACGGCCTCGCCCTG
OsBIERF3-BKT7-F	CGGAATTCATGCTGCTTAATCCGGCGTC
OsBIERF3-BKT7-R	CGGGATCCTTAGCTCACCAGCTGCTGGA
OsBIERF3-GEX-F	CGGGATCCATGCTGCTTAATCCGGCGTC
OsBIERF3-GEX-R	CGGAATTCTTAGCTCACCAGCTGCTGGA
Southern blotting	
HptII-Probe-F	ACACAGCCATCGGTCCAGAC
HptII-Probe-R	ATCTTAGCCAGACGAGCGGG
qRT-PCR	
OsBIERF3-RT-2F	AACTTCCCGCTCCGCATCG
OsBIERF3-RT-2R	GGATGAAGGGGAAGAAGAGCC
OsActin-F RT	AGCTGCGGGTATCCATGAGA
OsActin-R RT	GCAATGCCAGGGAACATAGTG
28S rDNA-F RT	TACGAGAGGAACCGCTCATTCAGATAATTA
28S rDNA-R RT	TCAGCAGATCGTAACGATAAAGCTACTC
eEF1 alpha-F RT	CAACCCTGACAAGATTCCCT
eEF1 alpha-R RT	AGTCAAGGTTGGTGGACCTC
OsPR3-RT-F	CACATACTGCGAGCCCAA
OsPR3-RT-R	TTGTAGGTGATCTGGATGGG
OsPR5-RT-F	TACAACGTCGCCATGAGCTTC
OsPR5-RT-R	TGGGCAGAAGACGACTTGGTAGTT
OsPR8-RT-F	TCTACGACGTGCAGAACAACTTCAG
OsPR8-RT-R	TCCAACTCAACCACTGTGCAAGTAA
OsPR10-RT-F	TGGTCCGGGCACCATCTAC
OsPR10-RT-R	CGAGCACATCCGACTTTAGG
Os02g41904-RT-F	TCCGCCTTCCTCCTCCTG
Os02g41904-RT-R	GCTCACGCACATGCCCTTG
Os01g62260-RT-F	TGGTGGACGGCTTCAACG
Os01g62260-RT-R	AGCTCGTCGGTGCGGAAG
Os06g47600-RT-F	GGCGTACTCCCGGCTGTT
Os06g47600-RT-R	CGGGCAGAAGGTGACGAT
Os12g38120-RT-F	TACAGCGTCAGCCTCGTGG
Os12g38120-RT-R	GGGCAGTCGCAGTTGAGGT
MPK3-RT-F	GACGCGAGGAAGTACATGAGG
MPK3-RT-R	CAGCGGGTTGAAGGTGAGC
MPK6-RT-F	TGACCCAAGCAGACGGATAACT
MPK6-RT-R	GCCAAGGATTCCCTCCAGAT
MEK3-RT-F	TGCTGCTGATATTTGGAGTCTTG
MEK3-RT-R	TCTTTTGGTGGTGTTGGTGATG
Os08g32600-RT-F	GAAGTAATTGTTGGGAGTGGGC
Os08g32600-RT-R	AGATGCTCTGGTATCGGTGGGT
Os12g16210-RT-F	AAAGGAGCACATGCAGCGTTAT
Os12g16210-RT-R	TCAGAGTCATCGAAAGGGTCAAA
Os10g19180-RT-F	GGTGTACTCCGCCACGTTTATT
Os10g19180-RT-R	AGCTAGTGTCCAGGGACTGATTT
Os10g39850-RT-F	GCTGCCTTCACTCCAGACGT
Os10g39850-RT-R	TCCTCGCTCAAGCATCCAA

Os02g44090-RT-F	GCACCGTCTGCTCCATGTG
Os02g44090-RT-R	GCCGTAGTAGTTGGTGCAGT
Os01g12860-RT-F	AGCACCACCCTACATGCCACA
Os01g12860-RT-R	TTCCTTCCGCCCTGGTCTATT
Os08g33660-RT-F	CGCCCTAGAATTATCCGAAAGC
Os08g33660-RT-R	CTGGGAACCATGACTCCTCAC
Os04g59260-RT-F	ACAACCAGGGCGTCTTCACC
Os04g59260-RT-R	TTCCTGTCAGTCCTCGGCAC
OsCesA7-F RT	TCCATCTTCTCCCTCGTCTG
OsCesA7-R RT	GAATCATCCATCCGGTCATC
OsCesA9-F RT	TTAGCACGTTTGCGAGTTTG
OsCesA9-R RT	GAACTCGTCGTCCTCGTCTC
Os10g20650-RT-F	AGGGAACGGTGGTGGGC
Os10g20650-RT-R	CCCGAAATCTCCTGGCAATC
Os03g12140-RT-F	GCCTTCTTCAACCGCTCCC
Os03g12140-RT-R	CGTCCACCGCCTCCTTGTT
